# Vector control with driving Y chromosomes: modelling the evolution of resistance

**DOI:** 10.1186/s12936-017-1932-7

**Published:** 2017-07-14

**Authors:** Andrea Beaghton, Pantelis John Beaghton, Austin Burt

**Affiliations:** Life Sciences, Imperial College, Silwood Park, Ascot, Berkshire, SL5 7PY UK

**Keywords:** Malaria, Gene drive, Resistance, *Anopheles gambiae*, Vector control, Driving Y chromosome, Branching process, Resistance

## Abstract

**Background:**

The introduction of new malaria control interventions has often led to the evolution of resistance, both of the parasite to new drugs and of the mosquito vector to new insecticides, compromising the efficacy of the interventions. Recent progress in molecular and population biology raises the possibility of new genetic-based interventions, and the potential for resistance to evolve against these should be considered. Here, population modelling is used to determine the main factors affecting the likelihood that resistance will evolve against a synthetic, nuclease-based driving Y chromosome that produces a male-biased sex ratio.

**Methods:**

A combination of deterministic differential equation models and stochastic analyses involving branching processes and Gillespie simulations is utilized to assess the probability that resistance evolves against a driving Y that otherwise is strong enough to eliminate the target population. The model considers resistance due to changes at the target site such that they are no longer cleaved by the nuclease, and due to trans-acting autosomal suppressor alleles.

**Results:**

The probability that resistance evolves increases with the mutation rate and the intrinsic rate of increase of the population, and decreases with the strength of drive and any pleiotropic fitness costs of the resistant allele. In seasonally varying environments, the time of release can also affect the probability of resistance evolving. Trans-acting suppressor alleles are more likely to suffer stochastic loss at low frequencies than target site resistant alleles.

**Conclusions:**

As with any other intervention, there is a risk that resistance will evolve to new genetic approaches to vector control, and steps should be taken to minimize this probability. Two design features that should help in this regard are to reduce the rate at which resistant mutations arise, and to target sequences such that if they do arise, they impose a significant fitness cost on the mosquito.

**Electronic supplementary material:**

The online version of this article (doi:10.1186/s12936-017-1932-7) contains supplementary material, which is available to authorized users.

## Background

Any attempt to control a harmful species, whether it be a disease-causing microbe or a crop-competing weed, must consider the potential for resistance to evolve—for example, to antibiotics or herbicides. The evolution of resistance has been a recurring theme over decades of malaria control efforts, both by the *Plasmodium* parasite to new drugs [1, 2] and by the *Anopheles* mosquito vector to new insecticides [3]. Any new intervention to control or help eliminate malaria must, therefore, be assessed in terms of how sustainable impacts are likely to be in the face of potential selection for resistance.

Recent progress in molecular and population biology has raised the possibility of new interventions for vector control using genetic approaches to disrupt the survival or reproduction of the mosquitoes, or to render them unable to transmit the parasite [4–7]. One possible approach is to make a synthetic driving Y chromosome by inserting, onto the Y, a gene encoding a nuclease that recognizes and cleaves a repeated sequence found only on the X chromosome, and have the appropriate control sequences such that the gene is only expressed during spermatogenesis [8–10]. The idea is that expression of the gene at this time will disrupt transmission of the X chromosome, leading to a preponderance of Y-bearing sperm and male offspring, which will themselves carry the nuclease gene. As long as the nuclease gene does not affect male fitness too strongly, the modified Y chromosome is expected to increase in frequency within a population, eventually replacing the wild-type Y. As it does so, the population sex ratio will become increasingly male-biased, which will have a direct impact in reducing disease transmission (because only females bite people and transmit disease). Since females are also likely to be most responsible for the productivity of the population, a male-biased sex ratio may also lead to a reduction in the total number of mosquitoes, further reducing transmission, and if the Y drive is sufficiently strong, then spread could lead to elimination of the population [9, 10]. Recently, there have been promising proof-of-principle demonstrations in *Anopheles gambiae* that cleavage of the X chromosome during spermatogenesis can lead to male-biased sex ratios with little or no effect on male fertility, both using engineered meganucleases [11] and with a CRISPR-based nuclease [12].

The spread of a driving Y may be expected to select for resistant genotypes. One obvious form of resistance would be changes in the target sequence such that it is no longer cleaved by the nuclease, as has been modelled and observed in the context of homing-based gene drive constructs [9, 10, 13–19]. In addition, since the spread of a driving Y will produce a male-biased sex ratio, there can be selection for autosomal suppressors that restore a 50:50 sex ratio [20, 21]. In the context of investigating a potential population modification (as opposed to population suppression) strategy for *Aedes* mosquitoes, Huang et al. [22] show using deterministic models that release of a driving Y can result in the spread of X-linked and autosomal resistance.

Though resistance to a driving Y may evolve, it is not inevitable. For example, resistant genotypes may arise sufficiently rarely that the population is eliminated by the driving Y before resistance evolves. Or resistance may have sufficiently large pleiotropic fitness effects that prevent it from spreading. To investigate further the likelihood that either target site resistance or a trans-acting suppressor will evolve to a driving Y, a population genetic and population dynamic model is developed. Stochastic effects are incorporated by extending the time-inhomogeneous branching process method of Uecker and Hermisson [23], which has also been used recently to analyse the evolution of resistance to homing-based gene drive elements that spread without causing population suppression [16]. Results are checked by fully stochastic Gillespie simulations [24]. The models identify a number of factors affecting the probability resistance evolves and rescues the population, including the mutation rate, the intrinsic rate of increase of the population, the strength of drive and the pleiotropic fitness costs of the resistant allele. In seasonally varying environments, the probability of resistance evolving is affected by the time of release of the driving Y males. Trans-acting suppressor alleles are more likely to suffer stochastic loss at low frequencies than target site resistant alleles.

## Methods

### Population biology and the driving Y

A continuous time differential equation model with separate sexes is developed, with explicit recruitment (birth) and death rates. Total recruitment rates depend on the number and fitness of females, under the assumption that males and fertilization are not limiting [25]. In such a model, the generation time is equal to the inverse of the death rate, and to keep this constant in the face of temporally variable population densities and environments, logistic density dependence is imposed on the recruitment rates (as a convenient way of modelling density-dependent mortality during the larval stage, which, for tractability, is not explicitly modelled here). Thus, the pre-release population model is:

1 $$\frac{dM\left( t \right)}{dt} = \left[ {R_{m} - \gamma N\left( t \right)} \right] F\left( t \right) - M\left( t \right)$$

$$\frac{dF\left( t \right)}{dt} = \left[ {R_{m} - \gamma N\left( t \right)} \right] F\left( t \right) - F\left( t \right)$$

where $M\left( t \right),\,F(t)$ and $N\left( t \right) = M\left( t \right) + F(t)$ are male, female, and total population numbers, *R*_*m*_ = *λ*/*μ* is the intrinsic growth rate of the population with density-independent birth rate *λ* and death rate *μ*, and *γ* is the density-dependent rate constant per generation time. Time is normalized by the average generation time 1/*μ*. In this model, the equilibrium total population size in the absence of control is $N_{0} = (R_{m} - 1)/\gamma .$ Definitions of parameters and variables for the model are given in Table 1.

**Table 1:** Description of model parameters and variables

Symbol	Definition
*R*_*m*_	Intrinsic growth rate of the population, *λ*/*μ*
*λ*	Density-independent birth rate
*μ*	Density-independent death rate
*γ*	Density-dependent rate constant per generation time
*m*	Proportion of progeny of driving Y males that inherit the driving Y
*u*	The fraction of female progeny of a driving Y male that inherit an X chromosome with a resistant mutation (Model I)
*v*	The chance of a suppressor mutation arising on an autosome (per individual per autosome, for all births, male or female) (Model II)
*w*	Fitness parameter for mutations (relative to fitness one for wild-types and driving Y males without the resistant gene). Model I: *w* for heterozygote females, $w^{2}$ for homozygous females and hemizygous males; Model II: *w* for heterozygotes, *w* ^2^ for homozygotes
*a*	Amplitude of seasonal variation in parameter for density-dependence, ${\upgamma }\left( t \right)$
*N*_0_	Equilibrium wild-type population size (before release of driving Y and in absence of mutation)
*h*_0_	Release amount of driving Y males
$H\left( t \right), M\left( t \right),F(t)$	Driving Y, wild-type male and wild-type female population sizes (non-mutant)
*N*(*t*)	Total population size
*P*_1_	Probability that at least one mutation arises and establishes, preventing population elimination
*P*_*Mut*_	Probability that at least one mutation arises (regardless of its fate)
*P*_*Con*_	Conditional probability that if one or more mutations arise, at least one establishes
*p*_*est*_(*t*_*a*_)	Probability that a single mutation arising at time *t*_*a*_ after release of the driving Y will establish (in the absence of other mutations)

Males with a driving Y chromosome are released into the population at time *t* = 0 in amount *h*_0_. The driving Y is assumed to have no effect on survival or mating success, and is transmitted to a proportion *m* of a male’s progeny, rather than the Mendelian 50%. Denoting the number of driving Y males at time *t* as *H*(*t*), the population dynamics are then captured by the following set of equations:

$$\frac{dH\left( t \right)}{dt} = 2[R_{m} - \gamma N\left( t \right)]\frac{mH\left( t \right)}{H\left( t \right) + M\left( t \right)}F\left( t \right) - H\left( t \right)$$

2 $$\frac{dM\left( t \right)}{dt} = [R_{m} - \gamma N\left( t \right)]\frac{M\left( t \right)}{H\left( t \right) + M\left( t \right)}F\left( t \right) - M\left( t \right)$$

$$\frac{dF\left( t \right)}{dt} = [R_{m} - \gamma N\left( t \right)]\frac{{M\left( t \right) + 2\left( {1 - m} \right)H\left( t \right)}}{H\left( t \right) + M\left( t \right)}F\left( t \right) - F\left( t \right)$$

As previously shown [25], there are two possible outcomes: either the sex ratio distortion is sufficient to eliminate the population (if $m > m_{crit} = 1 - 1/(2R_{m} )$) or else the driving Y goes to fixation, replacing the wild-type Y, and the population persists at a lower equilibrium density (here, equal to $N_{1} = [2(1 - m)R_{m} - 1]/[2(1 - m)\gamma ]$). In this paper, the focus is on the case where *m* is sufficiently high to eliminate the population (i.e., for the deterministic model, the population tends to zero as time goes to infinity), and the likelihood that resistance evolves and rescues the population before then is determined.

#### Model I: Target site resistance

For a driving Y chromosome that encodes a nuclease that recognizes and cuts a sequence on the X chromosome, the simplest form of resistance would be a change in the target site such that it is no longer recognized and cut by the nuclease. The *An. gambiae* sequences targeted by Galizi et al. [11, 12] are within the ribosomal DNA repeat, and are repeated hundreds of times on the X, but for simplicity in this initial analysis, it is supposed that there are only two types of X chromosomes possible, susceptible or fully resistant. It is further assumed that resistant alleles do not pre-exist in the population before release of the driving Y, nor do they arise spontaneously, but they do arise with probability *u* in the X-bearing gametes of driving Y males (i.e., they arise due to large-scale non-homologous repair of the cut sites, followed perhaps by some form of gene conversion or unequal crossing-over). Resistant mutations of this sort have previously been observed in yeast cells harbouring a meganuclease targeting their rDNA [26]. Also assumed is that the nuclease is only expressed during spermatogenesis, so mutant Xs arise singly, not in clusters, and that the occurrence of such a mutation does not affect the proportion of X-bearing sperm produced by a male—it is only when the mutant X occurs in subsequent generations with a driving Y that the resistance is manifest. There are, therefore, two types of Y and two types of X, giving four types of males and three types of females and a system of seven differential equations (Additional file 1: Eq. A1.2).

#### Model II: Trans-acting suppressor mutation

Also considered is the case of a trans-acting mutation on an autosome that suppresses the expression or activity of the nuclease. Such a mutation would not have a transmission advantage over the wild-type allele, but still can spread by natural selection, which favours a 50:50 sex ratio at autosomal loci [27]. It is unclear at this time how such a mutation might arise, and for simplicity it is assumed that it is fully dominant (i.e., there is complete suppression of the nuclease with only a single copy of the allele); that the mutation does not pre-exist in the population before release of the driving Y; and that it arises with probability *v* in all individuals, not just in the progeny of driving Y males. There are now six types of male and three types of female, and a system of nine differential equations (Additional file 1: Eq. A1.8).

### Stochastic methods

The deterministic equation models implicitly assume the population is effectively infinite, and so any mutation rate greater than zero ensures that resistant alleles will be created and, if sufficiently fit, get established and rescue the population. Any real population is finite, and therefore it is possible that the population is eliminated before a resistant mutation occurs, or if a mutation does occur before the population is eliminated, it may not establish due to stochastic loss. To estimate the probability that a resistant mutation arises and establishes before the population is eliminated, the branching process method first used in population genetics by Fisher [28] and Haldane [29] is applied. The more recent analysis by Uecker and Hermisson [23], applied by them to evolutionary rescue of a declining resident population by a single mutant type, is followed and extended to include differentiation between mutant types (males with/without the driving Y and females). It turns out that the linearization needed for the branching process model is only valid for a subset of the parameter space of interest, and fully stochastic Gillespie simulations (averaged over $10^{6}$ runs, *N*_0_ = 10^6^) are used to extend beyond this parameter space and to confirm results (see Additional file 1: Section A3, for details).

The main quantity of interest is the probability that at least one resistant (or suppressor) mutation arises and establishes in the population, preventing elimination. This is denoted as *P*_1_, and for Model I, where *u* is the fraction of female progeny of a driving Y male that inherit an X chromosome with a resistant mutation, the branching process yields:

3 $$P_{1} = 1 - { \exp }\left[ { - 2(1 - m)(uN_{0} ) \int \limits_{0}^{\infty } \frac{{p_{{est,F_{R} }} \left( \tau \right) \left( {R_{m} - \left( {R_{m} - 1} \right)N\left( \tau \right)} \right)F\left( \tau \right)H\left( \tau \right)}}{H\left( \tau \right) + M(\tau )}d\tau } \right]$$

Above, $p_{{est,F_{R} }} \left( \tau \right)$ is the probability that a single X chromosome mutation that arises at time $t_{a} = \tau$ in a daughter (denoted $F_{R}$) of a driving Y male will escape stochastic loss and establish; it is weighted by the rate at which new $F_{R}$ mutant individuals arise over time. The rate is a function of the non-mutant deterministic populations which are calculated from (2), in (3) shown normalized with *N*_0_ and with substitution of $\gamma = (R_{m} - 1)/N_{0 }$. Analogously for Model II for the trans-acting suppressor mutation, the following expression is obtained:

4 $$P_{1} = 1 - { \exp }\left[ { - 2(vN_{0} ) \int \limits_{0}^{\infty } \left( {R_{m} - (R_{m} - 1)N\left( \tau \right)} \right)F\left( \tau \right)\left( {p_{{est,F_{S} }} \left( \tau \right)\left[ {\frac{M\left( \tau \right) + 2(1 - m)H\left( \tau \right)}{H\left( \tau \right) + M\left( \tau \right)}} \right] + p_{{est,H_{S} }} \left( \tau \right)\left[ {\frac{2mH\left( \tau \right)}{H\left( \tau \right) + M\left( \tau \right)}} \right] + p_{{est,M_{S} }} \left( \tau \right)\left[ {\frac{M\left( \tau \right)}{H\left( \tau \right) + M\left( \tau \right)}} \right]} \right)d\tau } \right]$$

where *v* is the chance of a suppressor mutation arising on an autosome (per individual per autosome, for all births, male or female), and the integral term now includes probabilities of establishment of three mutant heterozygote types as *F*_*S*_, *M*_*S*_ and *H*_*S*_ (female, wild-type male and driving Y male mutants). For simpler systems with one mutant type, a closed-form integral solution for the probability of mutant establishment *p*_*est*_(*t*_*a*_) is possible using the Method of Characteristics [23, 30]; however, in general, analytical solutions are not possible for a Y drive model with different rates of transmission of the allele in different mutant types. Therefore, $p_{est, n} (t_{a} )$ is calculated for each mutant type $n$ by first deriving a partial differential equation for the probability generating function for the relevant multi-type branching process, and then employing the Method of Characteristics to transform the PDE into a set of coupled first order non-linear ordinary differential equations, one for each mutant type, that may be solved numerically to give each $p_{est, n} (t_{a} )$ [Additional file 1: Eq. A2.9 (Model I) and Eq. A2.15 (Model II)]. These may be substituted into *P*_1_ in (3) and (4) above [see Additional file 1: Sections A2.1 (Model I) and A2.2 (Model II) for further details].

To better understand the factors affecting this probability, results are also presented for two component probabilities: the probability that at least one resistant mutation arises before elimination, regardless of its fate (denoted *P*_*Mut*_), and the conditional probability that if one or more mutation arises, at least one survives stochastic loss and the population is rescued (denoted *P*_*Con*_). *P*_*Mut*_ is calculated for the resistant X-chromosome mutation (Model I) by integrating over the time-dependent population-wide rate at which $F_{R}$ mutant individuals arise (Additional file 1: Eq. A2.11), which yields:

5 $$P_{Mut} = 1 - { \exp }\left[ { - 2\left( {1 - m} \right)\left( {uN_{0} } \right)\mathop \int \limits_{0}^{\infty } \frac{{\left( {R_{m} - \left( {R_{m} - 1} \right)N\left( \tau \right)} \right)F\left( \tau \right)H\left( \tau \right)}}{H\left( \tau \right) + M\left( \tau \right)}d\tau } \right]$$

The non-mutant populations are calculated from (2), here again shown normalized by $N_{0}$. For trans-acting suppressor mutations (Model II), $P_{Mut}$ is calculated from the total rate that suppressor mutations arise in autosomes over all individuals (see Additional file 1: Eq. A2.16):

6 $$P_{Mut} = 1 - { \exp }\left[ { - 4(vN_{0} )\mathop \int \limits_{0}^{\infty } (R_{m} - (R_{m} - 1)N\left( \tau \right))F\left( \tau \right)d\tau } \right]$$

Finally, *P*_*Con*_, the conditional probability of establishment, is calculated as:

7 $$P_{Con} = \frac{{P_{1} }}{{ P_{Mut} }}$$

## Results

### Model I: Target site resistance

#### Cost-free resistance

**Fig. 1:**
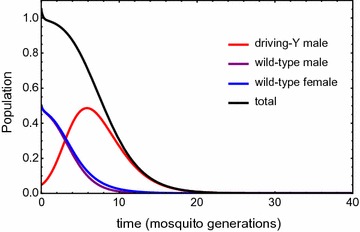
Example time course for population elimination for the deterministic model in the absence of mutation, after introduction of a driving Y chromosome at *t* = 0. Populations are normalized by the wild-type pre-release population *N*_0_, and parameter values are *R*_*m*_ = 6, *m* = 0.95, *h*_0_ = 0.05, *γ* = (*R*_*m*_ − 1)/*N*_0_

In the absence of resistance, release of a driving Y in this model leads either to population suppression or population elimination, depending on the transmission rate of the driving Y (*m*) and the intrinsic rate of increase of the population (*R*_*m*_). Assuming that *m* = 0.95 (consistent with the results of Galizi et al. [11, 12]), and *R*_*m*_ = 6 (consistent with the analyses of Deredec et al. [10]), then the model population tends to elimination, with time course calculated from (2) and shown in Fig. 1 (assuming an initial release of *h*_0_ = 0.05 *N*_0_, i.e. 5% of the pre-release equilibrium population size). As can be seen, the population size drops rapidly, such that it is only $\cong 30\%$ of its original size after 10 generations, and $\cong 0.2\%$ after 30 generations. The number of driving Y males initially increases due to the drive, and then decreases due to the reduction in total population size. The total number of driving Y males born over the time to elimination is $\cong 4\;N_{0}$, and the number of daughters of these males is $\cong 0.2\;N_{0}$ (see Additional file 1: Eq. A2.12). As it is assumed that mutations to target site resistance can only occur in the meiotic cells of driving Y males, and would only be transmitted to their daughters, these numbers give some indication of the opportunity for resistance to arise.

The stochastic model for the evolution of resistance requires one more parameter, the product of the initial population size (*N*_0_) and mutation parameter *u* (i.e., the fraction of female progeny of a driving Y male *H*(*t*) that inherit a mutant resistant X chromosome (0 ≤ *u* ≤ 1). If one considers the baseline value of *uN*_0_ = 1, then the probabilities of three possible outcomes can be calculated from the model: no resistant mutation arises and the population is eliminated ($1 - P_{Mut} \cong 80\%$ for baseline parameter values); at least one mutation does arise, but none establish, and the population is eliminated $(P_{Mut} - P_{1} \cong 13\% )$; and at least one mutation arises and establishes, and the population persists indefinitely $(P_{1} \cong 7\% ).$ The overall probability the population is eliminated is 93%. For these parameter values, the population is usually eliminated by the driving Y before a single resistant mutation occurs, and even when one does arise, most of the time the mutation is lost and the population is still eliminated. In the Additional file 1: Figure A3.1 presents some exemplar runs from the Gillespie simulations (10^6^ runs, *N*_0_ = 10^6^) for which at least one resistant mutation survives stochastic loss and the population recovers.

In those cases when resistance does evolve, it evolves quickly, and the number of females in the target population is suppressed for a relatively short time (e.g., it remains below 33 and 5% of its pre-release equilibrium value for 17.6 (median 17.3, interquartile range 16–19) and 12.3 (median 11.9, interquartile range 10.5–13.6) generations on average, respectively (full simulation model used, 10^5^ runs). To put these numbers into context, *An. gambiae* mosquitoes may have 10–18 generations per year, depending on temperature [31, 32]. As an aside, it is noted for comparison that if, for example, *m* = 0.9, which is insufficient to eliminate the target population, resistance will always evolve (assuming *u* > 0), and the population will be suppressed below 33% of its initial density for an average of 90.4 (median 62.5, interquartile range 28–122) generations before it recovers. It never goes below 5% of its initial value (detailed results in Additional file 1: Figure A3.2, Section A3).

In this model, there are three parameters that affect the probability that resistance evolves before the population is eliminated: the product of the mutation rate and population size (*uN*_0_), the intrinsic rate of increase of the population (*R*_*m*_), and the transmission rate of the driving Y (*m*). Now, the effect of varying each of these parameters individually on the probability of resistance evolving is investigated.

##### Varying *uN*_0_

From (5) in the limit of low *uN*_0_ ≪ 1, the probability that at least one mutation arises before elimination, *P*_*Mut*_, is proportional to *uN*_0_:

$$P_{Mut} \cong 2\left( {1 - m} \right)\left( {uN_{0} } \right)\mathop \int \limits_{0}^{\infty } \frac{{\left( {R_{m} - \left( {R_{m} - 1} \right)N\left( \tau \right)} \right)F\left( \tau \right)H\left( \tau \right)}}{H\left( \tau \right) + M(\tau )}d\tau$$

Since the probability that a single mutation establishes does not depend on *uN*_0_ in (3), the probability a mutation arises and establishes, allowing the population to persist (*P*_1_), is also proportional to *uN*_0_ for *uN*_0_ ≪ 1:

$$P_{1} \cong 2\left( {1 - m} \right)\left( {uN_{0} } \right)\mathop \int \limits_{0}^{\infty } \frac{{p_{{est,F_{R} }} \left( \tau \right)\left( {R_{m} - \left( {R_{m} - 1} \right)N\left( \tau \right)} \right)F\left( \tau \right)H\left( \tau \right)}}{H\left( \tau \right) + M(\tau )}d\tau$$

**Fig. 2:**
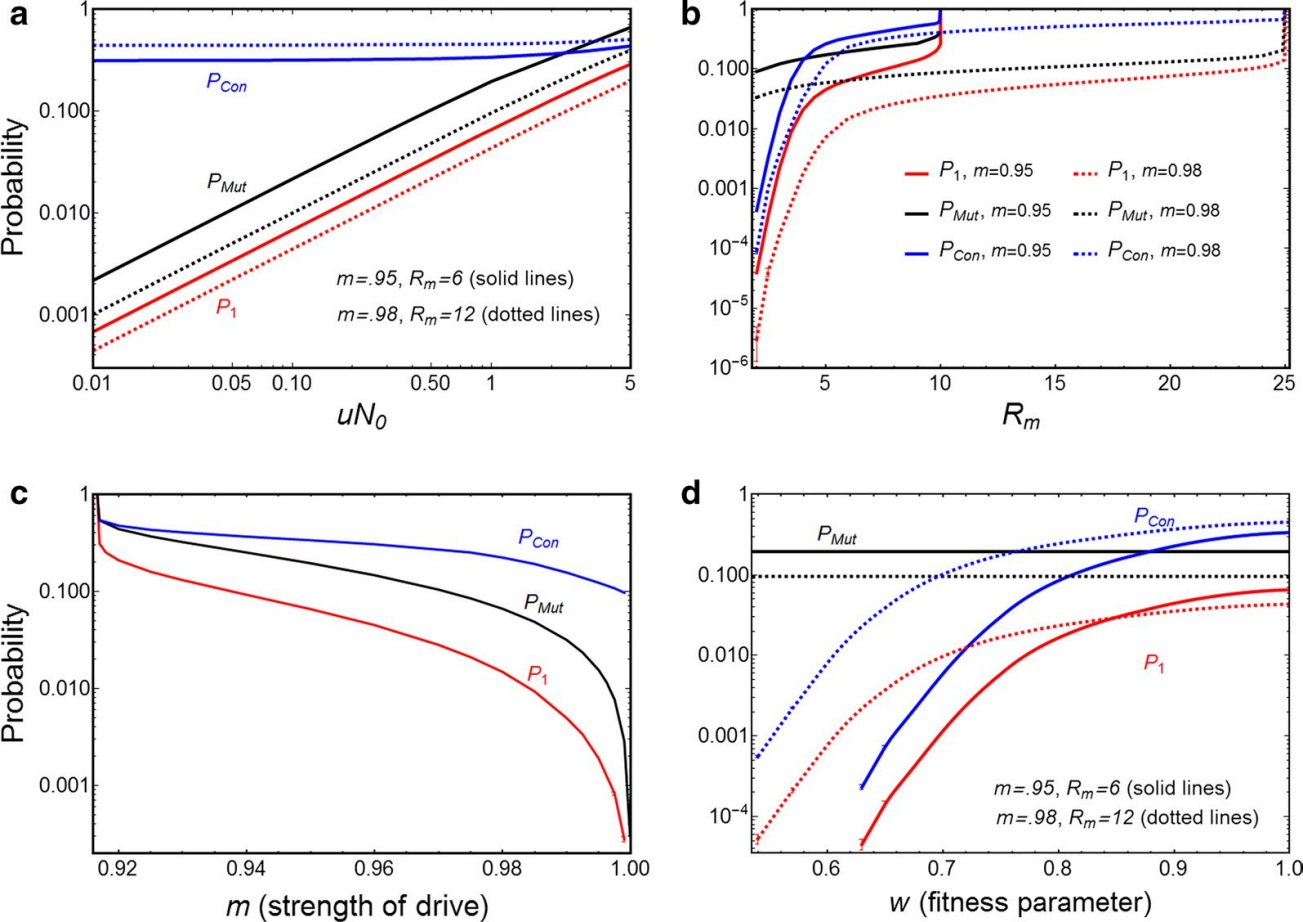
$P_{Mut}$ (*black lines*), *P*_1_ (*red lines*), and *P*_*con*_ (*blue lines*) for resistant cost-free mutations. **a** Probabilities $P_{1}$ and *P*_*Mut*_ increase with increasing *uN*_0_, for *R*_*m*_ = 6 and *m* = 0.95 (*solid lines*) or *R*_*m*_ = 12 and *m* = 0.98 (*dotted lines*). **b** All probabilities increase with increasing *R*_*m*_, for *m* = 0.95 (*solid lines*) and $m = 0.98$ (*dotted lines*), with *uN*_0_ = 1. Curves only extend as far as *R*_*m*_ = 1/[2(1 − *m*)(1 − *u*)] (see Additional file 1: end of Section A1.1b) above which the strength of Y drive *m* < *m*_*crit*_ and population elimination does not occur. **c** Probabilities decrease with increasing $m$ (strength of Y drive), for *R*_*m*_ = 6, *uN*_0_ = 1. **d** Probabilities increase with increasing mutant fitness parameter *w*, for $R_{m} = 6,m = 0.95$ (*solid lines*) and $R_{m} = 12,m = 0.98$ (*dotted lines*), and $uN_{0} = 1$. The deterministic model shows that the population will be eliminated for *w* ≤ 0.563 for *m* = 0.95, and for $w \le 0.452$ for *m* = 0.98. For all plots, *h*_0_ = 0.05. *Error bars* at low $R_{m} , w$ and high *m* show the standard error for simulations (averaged over $10^{6}$ runs, *N*_0_ = 10^6^), when the branching process model does not apply; if not shown, error is within thickness of plot line

This agrees with simulation results of Marshall et al. [19] for population-suppressing homing-based gene drive that show a linear relationship between $1/N_{0}$ and the cleavage-resistant allele generation rate leading to a given elimination probability. For sufficiently small values of *uN*_0_, the conditional probability therefore also does not vary with *uN*_0_, as expected due to the proportionality of both *P*_1_ and *P*_*Mut*_ on *uN*_0_, such that *uN*_0_ cancels out in (7). Figure 2a confirms these dependencies for varying $uN_{0} .$

##### Varying *R*_*m*_

Increasing *R*_*m*_ while keeping everything else the same leads to an increase in the population size, as $N_{0} = (R_{m} - 1)/\gamma$, and therefore in *uN*_0_, and consequently has much the same effects as an increase in *uN*_0_, as analysed above. Here it is asked, for populations of the same size but that differ in *R*_*m*_ (and therefore also differ compensatingly in *γ*), how are they expected to differ in the probability of evolving resistance? Increasing *R*_*m*_ in this way increases the time for a driving Y to eliminate the population, and therefore increases the opportunity for resistant mutations to arise before elimination (Fig. 2b, black lines). In addition, the probability that a mutation arising at a specified time $t_{a}$ becomes established (in the absence of others), *p*_*est*_(*t*_*a*_), increases as $R_{m}$ increases (Additional file 1: Section A2.1, Figure A2.2a). This is because at higher *R*_*m*_ the recruitment (birth) rate of new resistant mutants is higher, reducing the probability of stochastic loss. Since at higher *R*_*m*_ the probability of a mutation arising is higher, and its subsequent probability of surviving stochastic loss is also higher, the overall probability $P_{1}$ that resistance evolves increases with increasing *R*_*m*_ (red lines in Fig. 2b).

##### Varying *m*

Increasing the transmission rate of the driving Y (*m*) reduces the probability of at least one mutation arising before elimination (Fig. 2c, black lines). Again, there are several reasons for this. Firstly, there is a factor (1 − *m*) in the mutation rate because the proportion of mutant females born from driving Y males decreases according to the sex bias (1 − *m*) (Eq. 3). At the limit of *m* = 1, no mutations can arise at all because driving Y males only create other males. Secondly, with larger *m* there is less time for mutations to occur, since the driving Y eliminates the population more quickly. Close to *m*_*crit*_ (for these parameters, *m*_*crit*_ *=* 0.9167), the population is eliminated very slowly, providing more opportunity for a mutation to occur. The probability $p_{est} \left( {t_{a} } \right)$ that a single resistant mutation (that arises in a female at time *t*_*a*_) gets established in a population is also affected by *m* (Additional file 1: Figure A2.2b). So, with stronger Y drive, the probability that a resistant mutation occurs before elimination is lower, and if one does occur, the probability it establishes is lower, and thus the overall probability of resistance evolving is lower (Fig. 2c, red line).

#### Costly resistance

Thus far, it has been assumed that resistance is cost-free, with no pleiotropic effects on other fitness components. Now the case of resistance having a cost is considered. This is modelled as a decrease in fertility of females with the resistant gene, and decreased participation in mating for males with the gene (Additional file 1: Section A1.1). There are four genotypes carrying one or more resistant alleles, and the system of equations (Additional file 1: Eq. A1.2) has separate parameters for all of them. However, for simplicity of analysis here, these are collapsed to a single parameter, assuming that heterozygous females have fitness *w* < 1, and homozygous females and hemizygous males have fitness *w* ^2^ (relative to fitness one for wild-types and driving Y males without the resistant gene).

**Fig. 3:**
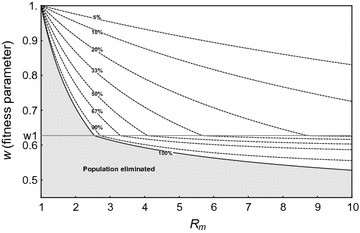
Deterministic equilibrium as a function of the fitness of the resistant mutation *w* (for heterozygous females, with fitness *w* ^2^ for homozygous females and hemizygous males) and the intrinsic rate of increase of the population (*R*_*m*_). The population is rescued in the white area, where dotted curves represent the percentage suppression of the total female population size, compared to its size when there is no fitness cost (*N*_0_/2). The population is eliminated in the shaded area under the curve of 100% population suppression (*w* = *w*_*ex*_, *solid black line*). For *w*_1_ = 0.627 < *w* ≤ 1, the resistant mutation tends to fixation, and in the white area the population is rescued (with reduced size equal to $\frac{{w^{2} R_{m} - 1}}{{w^{2} \gamma }}$), whereas in the shaded area, the population is eliminated. For 0 < *w* ≤ *w*_1_, the resistant mutation tends to an intermediate equilibrium, which again rescues the population only in the white area. For *R*_*m*_ ≥ 10.0001, the population is always nonzero, since the Y drive is not sufficient to eliminate the population. Parameters are *m* = 0.95, *u* = 10^−6^

To find the equilibria for a nonzero mutation rate, the time derivatives in the deterministic differential equations in the presence of mutation (Additional file 1: Eq. A1.2) are set to zero. Focussing initially on the deterministic model, Fig. 3 is a contour plot showing the fate of the resistant mutation and the resulting effect on the equilibrium number of females in the population as a function of *w* and *R*_*m*_, assuming *m* = 0.95 and *u* = 10^−6^ (see Additional file 1: Section A1.1b, for calculation of equilibria). The resistant allele fixes deterministically in the population for $w_{1} < w \le 1$, and establishes at intermediate equilibrium with the wild-type for 0 ≤ *w* < *w*_1_ (*w*_1_, which is independent of *R*_*m*_, is given in Additional file 1: Section A1.1b). The total population goes extinct (shaded area) if fitness is below *w* = *w*_*ex*_ (100% suppression of population, solid line), i.e. where net total population growth is not positive. Above *w* = *w*_*ex*_, the total population is nonzero and reduced, and the dotted contour lines in Fig. 3 show the percentage suppression of the total female population size, compared to its size when there is no fitness cost (*N*_0_/2). Therefore, a resistant mutation might establish, and even spread to fixation, but still not rescue the population if it is too costly. As expected, populations with higher *R*_*m*_ can be rescued by mutations with higher cost than populations with lower *R*_*m*_. Moreover, as the strength of Y drive $m$ is increased, the minimum fitness required for fixation of the mutation, $\,w_{1}$, decreases, as does *w*_*ex*_ (above *R*_*m*_ = 1/*w*_1_^2^), since the higher the Y drive, the more of an advantage the resistant allele has over the sensitive allele in transmitting to the next generation and in restoring the 50:50 sex ratio, and thus a higher fitness cost can be tolerated (Additional file 1: Figure A1.2). It is also found that there is very weak dependence of these results on the mutation parameter *u*, assuming it is low (*u* ≪ 1). Although the focus is on high *m* (strength of drive) for population suppression and elimination, it is possible to compare results for allele frequencies to the model of Huang et al. [22] for population modification using Y-linked drive and X-linked resistance. For *u* = 0, using the analytical expressions for allele frequencies for general fitnesses, a region of low *m* where the resistant X chromosome mutation cannot spread is similarly found, $m < 1 - \frac{{w_{{F_{R} }} w_{{H_{R} }} }}{{2(2 - w_{{F_{R} }} )}}$, which becomes the condition of Huang et al. (Equation (5), [22]) if their expressions for resistant heterozygote fitnesses are substituted.

Now the effect of fitness costs in the stochastic model is considered. Here, fitness costs do not affect the rate at which mutations arise (*P*_*Mut*_), which is constant, but do reduce the probability of surviving stochastic loss and ultimate establishment (*P*_1_, Fig. 2d). For high costs, the branching process cannot be used (as explained in Additional file 1: Section A2.3), and in this region full simulations are used. The conditional probability that at least one mutation survives stochastic loss if one or more mutations arise is also less for higher cost. Thus, higher fitness cost of the mutation (i.e., lower density-dependent recruitment rate for the heterozygotes) results in lower probability that a mutant mosquito will survive early stochastic loss, because it will be less able to pass on the allele before dying. In summary, the costlier resistance is (i.e., the lower *w*), the lower the probability that it will evolve, and if it does evolve, the lower the impact on the driving Y intervention.

#### Seasonal cycles

It has been shown above that a key parameter affecting the probability of resistance evolving is the initial population size, through the combined parameter *uN*_0_. In many locations, the number of mosquitoes shows dramatic fluctuations between wet and dry seasons [33, 34]. Previous theory has shown that the probability a beneficial mutation establishes and goes to fixation can be affected by such fluctuations, and can depend upon when in a seasonal cycle the mutation arises [35, 36]. Seasonality can also affect the time to elimination by a driving Y, depending on when in the cycle the releases are made [13]. To investigate the consequences of seasonal fluctuations in mosquito numbers for the evolution of resistance to a driving Y, periodic variation is incorporated into the model of mosquito demography via a sinusoidal time dependence in the parameter γ for density dependence in the mosquito recruitment rate, such that instead of being a constant, it varies seasonally:

8 $${\upgamma}\left( t \right) = {\upgamma}_{0} [a]\left( {1 + a\sin \left[ {\frac{2\pi t}{T}} \right]} \right)$$

Here, *a* is the amplitude of the oscillations in γ(*t*), and *T* is the seasonal period, in mosquito generations in (8) to be consistent with time *t*, which is normalized with generation time (1/*μ*). An increase in γ(*t*) (and thus reduction in recruitment rate) during the dry season could arise from a reduction in the number or productivity of breeding sites at this time. The effects of seasonality are investigated by varying the amplitude *a*, and as *a* is varied, γ_0_[*a*] is adjusted such that the mean population size over the cycle is kept constant (Additional file 1: Section A1.1a).

**Fig. 4:**
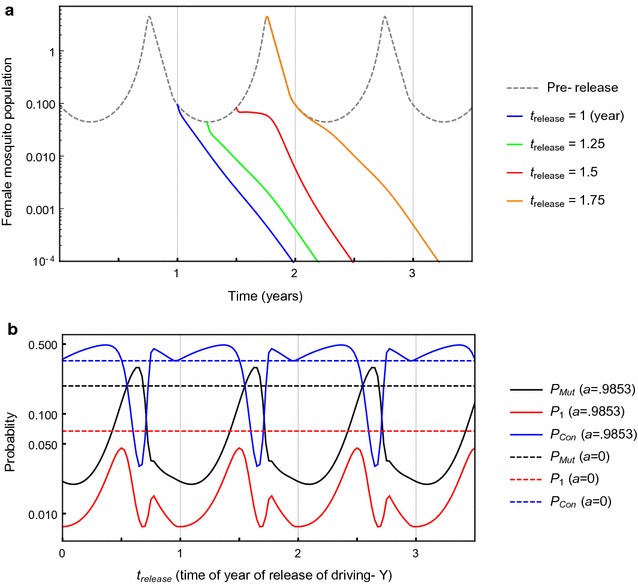
**a** Time evolution of wild-type female mosquito population *F*(*t*) before driving Y release (*dotted line*), and for introduction of the driving Y at time *T* (*blue line*), 1.25*T* (*green line*), 1.5*T* (*red line*), and 1.75*T* (*orange line*). For *R*_*m*_ = 6, m = 0.95, *T* = 18.25, *h*_0_ = 0.05, *w* = 1, *a* = 0.9825 (amplitude selected for peak to trough ratio of 100:1). Note *h*_0_ is the proportion of the time-averaged pre-release population size $\bar{N}\left( a \right) ( = N_{0} )$, so the absolute numbers released at different time points are assumed to be the same. **b** $P_{Mut}$, *P*_1_, and $P_{Con}$ as a function of $t_{release} ,$ the time when the driving Y is released during the period *T* of the seasonally-varying wild-type population, for *a* = 0.9825 (*solid lines*) and *a* = 0 (*dotted lines*; no seasonality). For parameters *R*_*m*_ = 6, m = 0.95, *h*_0_ = 0.05, *uN*_0_ = 1, *w* = 1, *T* = 18.25, and with ${\upgamma}_{0} \left[ a \right]$ adjusted as described as above, such that average wild-type populations over a period $\bar{N}\left( a \right) = N_{0}$

As an example, Fig. 4a shows the periodically-varying female population, with amplitude *a* chosen to give a peak/trough ratio of female mosquito numbers of 100:1, and a representative mosquito generation time of $1/\mu = 20$ days. Figure 4a also shows the deterministic female wild-type population for an initial amount of synthetic driving Y males, $h_{0 }$, introduced at different times of year. In Fig. 4a, the time of the first release (going into low season) is benchmarked as year one, with other sample releases at *t*_*release*_ *=* 3 months (population trough), 6 months (rising population) or 9 months (coincides with population peak) after year one. The female population (versus time) after introduction of the driving Y follows different paths to extinction depending on the time of year that the driving Y is released, *t*_*release*_. For no seasonality, for these parameters, it takes roughly one year (i.e. *T* = 18.25 mosquito generations) for the driving Y to crash the population to $\cong 1\%$ of its initial value for typical parameters.

Depending on the point in the yearly cycle that the driving Y is released, any mutation that then arises will encounter different changing conditions (i.e., population sizes and growth rates), which will in turn affect the probability that it will establish. In Fig. 4b, *P*_*Mut*_, *P*_1_ and $P_{con}$ are shown as functions of the times of synthetic driving Y release, *t*_*release*_, into the wild-type population during the year. The interplay between the yearly seasonal variation and dynamics of the driving Y establishment and extinction of the population strongly influences mutant creation and survival probabilities. The probability $P_{Mut}$ that a mutation arises (and subsequently may or may not survive) is highest when the driving Y is released at $t_{release} \cong 7.8$ months after each yearly benchmark in (Fig. 4b), which is well into the high season, so that subsequently the peak of the driving Y male coincides with the population peak and maximizes the rate of mutant creation. But mutants that are likely to have arisen during the population surge at high season subsequently experience periods of dropping recruitment rate, and thus decreased chance of the mutation establishing, so conversely $P_{con}$ is at its lowest for driving Y release at $t_{release} \cong 7.8$ months after each yearly benchmark.

**Fig. 5:**
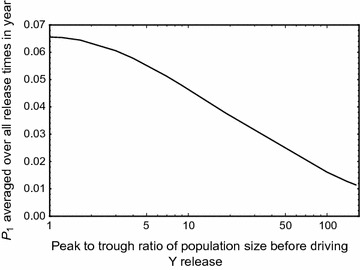
*P*_1,*avg*_, the probability of at least one mutation arising and surviving, averaged over all driving Y release times in a seasonal cycle *T* (1 year), as a function of the peak to trough ratio of the periodically-varying pre-release wild-type population (i.e., varying amplitude *a*). For *R*_*m*_ = 6, *m* = 0.95, *h*_0_ = 0.05, *uN*_0_ = 1, *w* = 1, *T* = 18.25 and ${\upgamma}_{0} \left[ a \right]$ adjusted such that average wild-type populations over a period are constant

The combination of these effects results in *P*_1_, the probability that at least one mutant will arise and survive, and the population will persist, being less for all release times for high seasonal variation (peak/trough ratio = 100:1) than for no seasonal variation, and at two times of the year (just before the population peak and just before the trough), it is approximately tenfold less. For low-amplitude seasonal variation (not shown), there are some individual release times in the year for which *P*_1_ is greater than in the equivalent non-seasonal model; however, *P*_1_ averaged over all times of driving Y release is always less than for the model with no seasonality for *all* amplitudes of variation (Fig. 5). Thus, comparing populations with the same mean population size, seasonal variation decreases the overall chance of successful mutation and population rescue (when averaged equally over all possible release times).

### Model II: Trans-acting suppressor

#### Cost-free suppression

Now the possibility is considered that a mutation arises on an autosome that suppresses the expression or activity of the driving Y. As before, to analyse the fate of new mutations, a combination of a branching process model and Gillespie stochastic simulations is used (averaged over $10^{6}$ runs) (Additional file 1: Section A3). Here mutations can arise in all individuals, not just the daughters of driving Y males, though for simplicity it is still assumed that no mutations exist before release. *v* (0 ≤ *v* ≤ 1) is defined as the chance of the suppressor mutation arising on the relevant autosome (per individual per autosome, for all births, male or female).

With baseline parameters of *R*_*m*_ = 6, and *m* = 0.95, $\sim 7.3$ *N*_0_ individuals are born between release of the driving Y and elimination of the population (see explanation after Additional file 1: Eq. A2.17); and so there are more individuals in which suppressor mutations can arise than for target site resistance mutations. However, the probability of a new suppressor mutation surviving stochastic loss is less than for a resistant mutation at all times of arising [compare *p*_*est*_(*t*_*a*_) in Additional file 1: Figures A2.1b (Model I) and A2.4b (Model II)]. A contributing factor is that the suppressor is on an autosome rather than on the X-chromosome: firstly, new suppressor mutations arise in males and females rather than only in females, and secondly, mutant males *H*_*S*_ and *M*_*S*_ pass the suppressor mutation equally to males and females while $H_{R}$ and $M_{R}$ pass on the resistant X-chromosome at the same rate but only to females. Consequently, when the mutation is rare, the proportion of time spent in a male vs female mutant is higher for the suppressor mutation than for the X-chromosome mutation, and since mutant males have a lower probability of stochastic survival than females (see Additional file 1: Figure A2.4b), early suppressor mutant populations are overall less likely to survive than resistant ones. Despite the lower probability of each mutation establishing, the greater opportunity for mutations to arise means that for equal mutation rates (i.e., *u* = *v*), a suppressor is more likely to establish than a target site resistance allele. With $v\varvec{N}_{0}$ = 1 and for baseline parameters, $P_{Mut} \cong 1$ and $P_{1} \cong 0.6.$

**Fig. 6:**
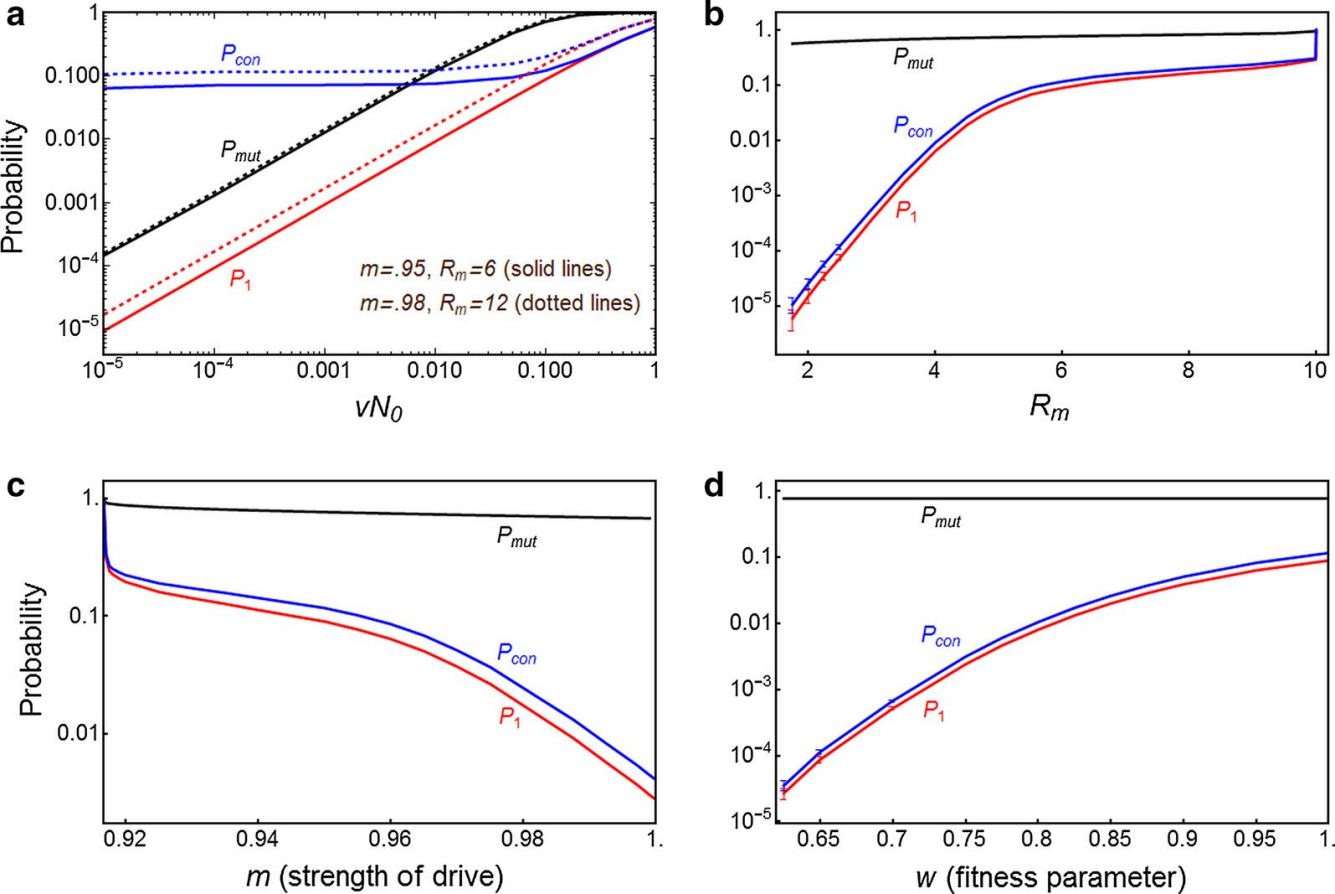
$P_{Mut}$ (*black line*), $P_{1}$ (*red line*) and *P*_*Con*_ (*blue line*) for the trans-acting suppressor mutation. **a** Probabilities increase with increasing *vN*_0_ for *R*_*m*_ = 6, *m* = 0.95 (*solid lines*) and for *R*_*m*_ = 12, *m* = 0.98 (*dotted lines*), and *w* = 1. **b** Probabilities increase with increased intrinsic growth rate *R*_*m*_ (for *m* = 0.95, *w* = 1, *vN*_0_ = 0.1). **c** Probabilities decrease with increased Y-drive *m* (for *R*_*m*_ = 6, *w* = 1, *vN*_0_ = 0.1. For all plots, *h*_0_ = 0.05. **d** Probabilities decrease with lower fitness $w$ (for *R*_*m*_ = 6, *m* = 0.95). The deterministic model shows that the population will be eliminated for *w* ≤ 0.61. For all plots, *h*_0_ = 0.05, *vN*_0_ = 0.1. The* error bars* at low *R*_*m*_ and *w* show the standard error for simulations (averaged over $10^{6}$ runs, *N*_0_ = 10^6^), when the branching process model does not apply; if not shown, error is within thickness of plot line

The effect of varying the underlying parameters $v\varvec{N}_{0}$, *R*_*m*_, and *m* is now considered. The effects are much like those for the target site resistance model, and for much the same reasons, though quantitative details differ. From (6) in the limit of low *uN*_0_ ≪ 1, *P*_*Mut*_ is proportional to $v\varvec{N}_{{0\varvec{ }}}$, $P_{Con}$ is largely independent of $v\varvec{N}_{0}$, and therefore *P*_1_ is proportional to $v\varvec{N}_{0}$. These results are shown for baseline parameters in Fig. 6a. As *R*_*m*_ increases (while keeping *N*_0_ constant), the time to eliminate the population increases, giving more opportunity for suppressor mutations to arise, and if they do arise, then they have a higher probability of establishing because low-density recruitment rates are higher, resulting in a higher overall probability of a suppressor establishing (Fig. 6b). As the transmission rate of the driving Y (*m*) increases, the time to elimination decreases, and therefore the opportunity for a suppressor to arise decreases. A difference from the target site resistance model is that even for *m* = 1, mutations can arise in the suppressor model, while for the previous model, mutations only arise in females fathered by driving Y males, and therefore none can arise for a 100% male sex bias. The conditional probability of a mutation surviving also decreases with increasing *m*, and therefore the overall probability *P*_1_ also decreases (Fig. 6c).

#### Costly suppression

The case of the suppressor allele having a cost is now considered. For simplicity, it is assumed that heterozygous females and males have fitness *w* < 1, and homozygous females and males have fitness *w* ^2^.

**Fig. 7:**
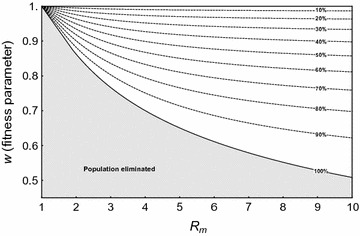
The percentage suppression of the total female population (*dotted lines*) as a function of the fitness of the resistant suppressor mutation (*w* for heterozygotes, *w* ^2^ for homozygotes) and the intrinsic rate of increase of the population (*R*_*m*_). The solid line shows the extinction curve *w* = *w*_*ex*_ (100% suppression line), below which the population is eliminated (*shaded area*). For $1 \ge w > w_{1} = \left( {1 - 10^{ - 7} } \right)$ (zone too narrow to appear on plot), the suppressor autosome tends to fixation; below *w* = *w*_1_ (all visible areas of plot) it tends to an intermediate equilibrium, which rescues the population only in the *non-shaded area*. For *m* = 0.95, *v* = 10^−7^

Focussing firstly on the deterministic model, Fig. 7 is a contour plot showing the fate of a suppressor and the population as a function of *w* and *R*_*m*_ (see Additional file 1: Section A1.2a, for calculation of equilibria from the system of deterministic equations). With a fitness cost to the suppressor mutation, one difference to the resistant model is that the region where the allele with the suppressor mutation is fixed, $1 > w \ge w_{1} = 1 - v$, is extremely narrow for *v* ≪ 1 and is not discernible on Fig. 7 with baseline parameters (*m* = 0.95, *v* = 10^−7^). Below $w_{1}$ (all visible areas on the plot), the suppressor allele is at intermediate equilibrium with the wild-type allele. Also shown are curves of constant percentage suppression of the total female population (dotted lines). The population is eliminated in the shaded area below the 100% extinction line *w* = *w*_*ex*_. There is little dependence of the results on $v$ for *v* ≪ 1, and an insignificant decrease in *w*_1_ and *w*_*ex*_ with increasing Y-drive *m* (not shown). Thus again, unless fitness cost to the mutation is too high and/or *R*_*m*_ is low, a suppressor mutation may establish and spread, leading to population rescue.

Note that trans-acting suppressors are less tolerant of fitness costs than target site resistant alleles (e.g., the shaded area is larger in Fig. 7 than in Fig. 3). This is because the resistant X-chromosome has a direct transmission advantage compared to sensitive alleles in the presence of the driving Y [passed to half rather than (1 − *m*) of offspring from a driving Y male]. The autosomal allele has no such transmission advantage over the wild-type, although in both cases the mutation restores the 50:50 sex ratio. This difference also fits with the already noted difference that suppressors have a lower probability of establishing than target site resistant alleles. Finally, as expected, increasing the fitness cost of a suppressor reduces the probability of it surviving stochastic loss and establishing (Fig. 6d).

## Discussion

This paper has considered driving Y chromosomes that are capable of eliminating a closed, random-mating population, and modelled the factors affecting the probability that resistance evolves before that happens. This topic of ‘evolutionary rescue’ has previously been studied in the context of populations threatened by a change in the external environment [23, 37–41]; the key differences here are that the risk to the population is a driving Y, with its own particular dynamics, and whose mechanism of action can itself give rise to one form of resistance. The models have identified several factors affecting the probability resistance evolves. Some are properties of the target population, in particular the size and the intrinsic rate of increase; all else being equal, higher values of both these parameters make resistance more likely. Other parameters are properties of the driving Y, and highlight two primary routes to minimizing the risks of resistance [9]. The first strategy is to target essential sites such that resistant alleles are likely to have pleiotropic fitness costs (previously analysed in the context of homing-based gene drive construct [9, 10, 13, 16]). This factor makes the rDNA repeat a more attractive target than some less important or nonfunctional repeat on the X chromosome; targeting functional sites within the rDNA repeat may also be better than targeting nonfunctional sites in the same repeat, though a fuller description of the mutations caused by rDNA-targeting nucleases would be helpful here. The second strategy is to ensure the mutation rate to resistance is low. In the context of a nuclease-based driving Y, targeting a sequence present in hundreds of copies will be better in this regard than targeting a single copy sequence (which may not lead to preferential inheritance of the Y in any case). Presumably $u$ could be further lowered by targeting a second sequence within the rDNA repeat. With no obvious limit to the number of sites that can be targeted in the rDNA, management of target site resistance would seem to be achievable.

The modelling has also revealed two other factors that can reduce the probability of resistance evolving. Increasing the transmission rate of the driving Y (*m*) reduces the opportunity for resistant mutations to arise. Note, though, in spatial models without resistance, suppression may be maximised at an intermediate optimum *m* [13, 42]. Releasing the construct at the right time of year can also reduce the probability of resistance evolving. In this model, this occurred just before the peak of abundance and also just as it was entering the trough, but other models of population fluctuation and other release rates ought to be examined. Note too that if releases are made in one location with the intention that the construct spread to other locations, one will have little control over when those migration events occur.

The evolution of trans-acting suppressors was also briefly considered. It is difficult to predict the most likely molecular mechanisms for such suppressors, and in the absence of a clear expectation, a simple model is considered in which mutations do not pre-exist in population before release, but can arise in any individual, not just in progeny of driving Y males. Pre-existing mutations could be included in the analysis using the approach of Hermisson and Pennings [43]; see also [16]. It is also possible to imagine ways that a suppressor mutation might arise from the nuclease gene, which would only be possible in the progeny of driving Y males—for example, a duplicated or retro-transposed copy of the nuclease gene that interferes with the original gene at the RNA level (e.g. RNAi), or at the protein level (e.g., competitive binding of a non-active enzyme).

Several of the modelling assumptions used here are worth highlighting. First, it is assumed that resistance is all-or-nothing. This is a reasonable first step, but partial target site resistance may occur if the target site is present in hundreds of copies, as in the rDNA repeat. Indeed, the action of the nuclease would likely lead to many different alleles being produced, with different degrees of resistance and different pleiotropic fitness effects. The overall probability of resistance evolving would then be a sum across possible alleles of *P*_1_ calculated for each allele, where *P*_1_ for the *i*th allele would depend on the mutation rate *u*_*i*_ and fitness effects *w*_*i*_ for that allele. Ideally, for all possible mutations, either the fitness, the mutation rate, or the degree of resistance provided is sufficiently low as to have a low probability of rescuing the population. Unfortunately, there is likely to be a limit to how thoroughly one will be able to test for resistance and suppressors in the lab, before release in the field. If high fitness resistance is seen in the lab, it will likely arise in the field, but the failure to see it in the lab will not guarantee it will not arise in the field.

It is also assumed that the driving Y has no fitness effects except the sex ratio distortion, so its frequency increases monotonically in the deterministic model as long as there are any susceptible genotypes remaining in the population (i.e., there is no complex dynamics, such as cycling [22, 44]). In the context of evolution of resistance, the main impact of a driving Y that has a cost on survival or mating success would likely be to slow down the spread of the driving Y and therefore slightly increase the probability of resistance evolving. A further assumption is that the daughters of driving Y males, which could harbour resistant mutations, have normal fitness. Galizi et al. [11] found that the ~5% of females produced had low fitness, presumably due to disruption of the rDNA. If the same were true of resistant types (e.g., they were also missing many rDNA repeats), then the effective transmission of the Y chromosome could be closer to one, reducing the probability of resistance evolving. It is further assumed that each offspring is derived from an independent mating, rather than, as usual with *Anopheles gambiae*, females mating only once in their life; incorporating this effect into the model would increase the amount of stochasticity, and reduce the probability of resistance evolving.

Finally, another assumption is that the population is closed and random-mating, whereas real *An. gambiae* populations exist over a landscape. Previous modelling has used a range of approaches to investigate the spatial spread of a driving Y [13, 25, 42], and these models should be extended to investigate the evolution of resistance. Some insight into the likely dynamics can be gained even from the current model. For example, consider a landscape of, say, 10,000 patches, each of which individually is a randomly mating population. If a driving Y is released into all of them, and $P_{1 } = 0.001$ for each patch, then it would be expected to spread and eliminate 9990 patches, and for resistance to evolve in ten of them; depending on how mosquitoes move on the landscape, those resistant types could eventually spread out from those patches and recolonize the landscape. The duration of protection offered by the driving Y will then vary from patch to patch, being shortest in those patches where the resistance first evolves, and longest in the last patch to be recolonized. If the patches vary, the modelling suggests resistance is more likely to arise in patches with a higher density of mosquitoes and, separately, that have a higher *R*_*m*_; these could act as source populations for the others. An interesting precedent in this regard is given by the evolution of insecticide resistance, in which the same nucleotide change has arisen and established multiple times in different genetic backgrounds [45], which implies that the (continental scale) population size of *An. gambiae* is larger than the inverse of the nucleotide mutation rate [46]. Note that on a landscape, it may be difficult for multiple resistant types to establish. In this example, if there is simultaneous release of a second construct that is sufficiently different from the first that resistance to one would not provide resistance to the other, then resistance to the second may also arise in ten patches, but if they are a different ten patches, then there may be no opportunity for the multiply-resistant type to evolve, and the population could be eliminated across the landscape. But even in the absence of resistance, there can be complex landscape dynamics between local elimination and recolonization [13, 42] which warrant further analysis.

It will be worthwhile investigating the interactions between genetic technologies and other interventions more broadly. There is an automatic synergy between different interventions that reduce population size in terms of reducing the probability of resistance evolving: anything that reduces population size, like bed nets or IRS, will reduce the probability that resistance evolves to a driving Y. Likewise, release of a driving Y will likely reduce the probability that resistance evolves to new insecticides, or even to new anti-malarial drugs.

## Conclusions

As with any other form of vector control, it is important to consider how the effectiveness of novel genetic approaches may be limited by the evolution of resistance. Previous studies have examined the sustainability of strategies using the homing reaction to a target population [14–16] or eliminate it [9, 10, 13]; here, the likelihood that resistance to a nuclease-based population-suppressing driving Y evolves before elimination is investigated. The modelling has demonstrated that resistance is more likely to evolve if the target population is large and has a high intrinsic rate of increase. The probability of resistance can be minimized by releasing insects carrying constructs for which mutations having a substantial effect on the rate of drive are unlikely to arise and/or have large pleiotropic fitness costs.

## Additional file

**Additional file 1.** Description of model, methods and supplementary results. **Section A1.** describes the deterministic differential equations for the genotype populations for each model and the derivation of the equilibria, for both the X chromosome resistant mutation (Model I) and the trans-acting suppressor mutation (Model II). **Section A2**. shows the application of the branching process method for calculating the probability of stochastic loss of new mutations, and the parameter space of applicability of the method is indicated. **Section A3**. contains details of the Gillespie stochastic simulations, used for parameter spaces where the branching process method does not apply, and to confirm results; effects of finite size of wildtype populations in simulations are discussed. In each section, supplementary results are shown: Figure A1.1, time-course for the deterministic equations for cost-free mutation (Model I); Figure A1.2, outcome of equilibria for a range of values of the strength of drive *m* (Model I); Figure A1.3, time-course for the deterministic equations for cost-free mutation (Model II); Figure A2.1, evolution in time of the mutation rate and probabilities of mutation and stochastic survival (Model I); Figure A2.2, probability of establishment of a single cost-free resistant mutation as a function of intrinsic growth rate *R*_*m*_ and strength of drive *m* (Model I); Figure A2.3, time evolution of the population-wide rates that new mutations arise (Model II); Figure A2.4, time evolution of mutation rates and probabilities of mutation and stochastic survival (Model II); Figure A3.1, recovery of the total female population due to establishment of resistant mutation in X chromosome (Model I); Figure A3.2, times of suppression of the total female population below its pre-driving-Y release value, before it is rescued by a resistant mutation (Model I).

## References

[1] White NJ (2004). Antimalarial drug resistance. J Clin Invest.

[2] Cui L, Mharakurwa S, Ndiaye D, Rathod PK, Rosenthal PJ (2015). Antimalarial drug resistance: literature review and activities and findings of the ICEMR network. Am J Trop Med Hyg.

[3] Barnes KG, Weedall GD, Ndula M, Irving H, Mzihalowa T, Hemingway J (2017). Genomic footprints of selective sweeps from metabolic resistance to pyrethroids in African malaria vectors are driven by scale up of insecticide-based vector control. PLoS Genet.

[4] Alphey L, McKemey A, Nimmo D, Neira Oviedo M, Lacroix R, Matzen K (2013). Genetic control of *Aedes* mosquitoes. Pathog Glob Health.

[5] Alphey L (2014). Genetic control of mosquitoes. Annu Rev Entomol.

[6] Burt A (2014). Heritable strategies for controlling insect vectors of disease. Philos Trans R Soc Lond B Biol Sci.

[7] Adelman ZN, Tu Z (2016). Control of mosquito-borne infectious diseases: sex and gene drive. Trends Parasitol.

[8] Burt A (2003). Site-specific selfish genes as tools for the control and genetic engineering of natural populations. Proc Roy Soc Lond B.

[9] Deredec A, Burt A, Godfray HCJ (2008). The population genetics of using homing endonuclease genes in vector and pest management. Genetics.

[10] Deredec A, Godfray HCJ, Burt A (2011). Requirements for effective malaria control with homing endonuclease genes. Proc Natl Acad Sci USA.

[11] Galizi R, Doyle LA, Menichelli M, Bernardini F, Deredec A, Burt A (2014). A synthetic sex ratio distortion system for the control of the human malaria mosquito. Nat Commun.

[12] Galizi R, Hammond A, Kyrou K, Taxiarchi C, Bernardini F, O’Loughlin S (2016). A CRISPR-Cas9 sex-ratio distortion system for genetic control. Sci Rep.

[13] Eckhoff PA, Wenger EA, Godfray HCJ, Burt A (2017). Impact of mosquito gene drive on malaria elimination in a computational model with explicit spatial and temporal dynamics. Proc Natl Acad Sci USA.

[14] Beaghton A, Hammond A, Nolan T, Crisanti A, Godfray HCJ, Burt A (2017). Requirements for driving anti-pathogen effector genes into populations of disease vectors by homing. Genetics.

[15] Noble C, Olejarz J, Esvelt KM, Church GM, Nowak MA (2017). Evolutionary dynamics of CRISPR gene drives. Sci Adv.

[16] Unckless RL, Clark AG, Messer PW (2017). Evolution of resistance against CRISPR/Cas9 gene drive. Genetics.

[17] Hammond AM, Kyrou K, Bruttini M, North A, Galizi R, Karlsson X (2017). The creation and selection of mutations resistant to a gene drive over multiple generations in the malaria mosquito. bioRxiv.

[18] Champer J, Reeves R, Oh SY, Liu C, Liu J, Clark AG (2017). Novel CRISPR/Cas9 gene drive constructs in *Drosophila* reveal insights into mechanisms of resistance allele formation and drive efficiency in genetically diverse populations. bioRxiv.

[19] Marshall JM, Buchman A, Sánchez CHM, Akbari OS (2017). Overcoming evolved resistance to population-suppressing homing-based gene drives. Sci Rep.

[20] Vaz SC, Carvalho AB (2004). Evolution of autosomal suppression of the *sex*-*ratio* trait in Drosophila. Genetics.

[21] Burt A, Trivers R (2006). Genes in conflict: the biology of selfish genetic elements.

[22] Huang Y, Magori K, Lloyd AL, Gould F (2007). Introducing desirable transgenes into insect populations using Y-linked meiotic drive—a theoretical assessment. Evolution.

[23] Uecker H, Hermisson J (2011). On the fixation process of a beneficial mutation in a variable environment. Genetics.

[24] Gillespie DT (2007). Stochastic simulation of chemical kinetics. Annu Rev Phys Chem.

[25] Beaghton A, Beaghton PJ, Burt A (2016). Gene drive through a landscape: reaction-diffusion models of population suppression and elimination by a sex ratio distorter. Theor Popul Biol.

[26] Muscarella DE, Vogt VM (1993). A mobile group I intron from *Physarum polycephalum* can insert itself and induce point mutations in the nuclear ribosomal DNA of *Saccharomyces cerevisiae*. Mol Cell Biol.

[27] Fisher RA (1930). The genetical theory of natural selection.

[28] Fisher RA (1922). On the dominance ratio. Proc R Soc Edinb.

[29] Haldane JBS (1927). A mathematical theory of natural and artificial selection. V. Selection and mutation. Proc Camb Philos Soc..

[30] Allen LJS (2010). An introduction to stochastic processes with applications to biology.

[31] Depinay JMO, Mbogo CM, Killeen G, Knols B, Beier J, Carlson J (2004). A simulation model of African Anopheles ecology and population dynamics for the analysis of malaria transmission. Malar J.

[32] Mordecai EA, Paaijmans KP, Johnson LR, Balzer C, Ben-Horin T, de Moor E (2013). Optimal temperature for malaria transmission is dramatically lower than previously predicted. Ecol Lett.

[33] Amek N, Bayoh N, Hamel M, Lindblade KA, Gimnig JE, Odhiambo F (2012). Spatial and temporal dynamics of malaria transmission in rural Western Kenya. Parasit Vectors.

[34] Lehmann T, Dao A, Yaro AS, Diallo M, Timbiné S, Huestis DL (2014). Seasonal variation in spatial distributions of *Anopheles gambiae* in a Sahelian village: evidence for aestivation. J Med Entomol.

[35] Ewens WJ (1967). The probability of survival of a new mutant in a fluctuating environment. Heredity.

[36] Otto SP, Whitlock MC (1997). The probability of fixation in populations of changing size. Genetics.

[37] Bell G, Gonzalez A (2009). Evolutionary rescue can prevent extinction following environmental change. Ecol Lett.

[38] Gonzalez A, Ronce O, Ferriere R, Hochberg ME (2013). Evolutionary rescue: an emerging focus at the intersection between ecology and evolution. Philos Trans R Soc Lond B Biol Sci.

[39] Martin G, Aguilée R, Ramsayer J, Kaltz O, Ronce O (2013). The probability of evolutionary rescue: towards a quantitative comparison between theory and evolution experiments. Philos Trans R Soc Lond B Biol Sci.

[40] Orr H, Unckless R (2014). The population genetics of evolutionary rescue. PLoS Genet.

[41] Uecker H, Otto SP, Hermisson J (2014). Evolutionary rescue in structured populations. Am Nat.

[42] North A, Burt A, Godfray HCJ (2013). Modelling the spatial spread of a homing endonuclease gene in a mosquito population. J Appl Ecol.

[43] Hermisson J, Pennings PS (2005). Soft sweeps: molecular population genetics of adaptation from standing genetic variation. Genetics.

[44] Hall DW (2004). Meiotic drive and sex chromosome cycling. Evolution.

[45] Miles A, Harding NJ, Botta G, Clarkson C, Antao T, Kozak K (2016). Natural diversity of the malaria vector *Anopheles gambiae*. bioRxiv.

[46] Karasov T, Messer PW, Petrov DA (2010). Evidence that adaptation in *Drosophila* is not limited by mutation at single sites. PLoS Genet.

