# Supplementary material for: Vector control with driving Y chromosomes: modelling the evolution of resistance
Source: Malar J. 2017 Jul 14;16:286. doi: 10.1186/s12936-017-1932-7 (PMC5513332; doi:10.1186/s12936-017-1932-7)
Supplement: Supplementary file 1 — Additional file 1. Description of model, methods and supplementary results. Section A1. describes the deterministic differential equations for the genotype populations for each model and the derivation of the equilibria, for both the X chromosome resistant mutation (Model I) and the trans-acting suppressor mutation (Model II). Section A2. shows the application of the branching process method for calculating the probability of stochastic loss of new mutations, and the parameter space of applicability of the method is indicated. Section A3. contains details of the Gillespie stochastic simulations, used for parameter spaces where the branching process method does not apply, and to confirm results; effects of finite size of wildtype populations in simulations are discussed. In each section, supplementary results are shown: Figure A1.1, time-course for the deterministic equations for cost-free mutation (Model I); Figure A1.2, outcome of equilibria for a range of values of the strength of drive m (Model I); Figure A1.3, time-course for the deterministic equations for cost-free mutation (Model II); Figure A2.1, evolution in time of the mutation rate and probabilities of mutation and stochastic survival (Model I); Figure A2.2, probability of establishment of a single cost-free resistant mutation as a function of intrinsic growth rate R m and strength of drive m (Model I); Figure A2.3, time evolution of the population-wide rates that new mutations arise (Model II); Figure A2.4, time evolution of mutation rates and probabilities of mutation and stochastic survival (Model II); Figure A3.1, recovery of the total female population due to establishment of resistant mutation in X chromosome (Model I); Figure A3.2, times of suppression of the total female population below its pre-driving-Y release value, before it is rescued by a resistant mutation (Model I). [file 12936_2017_1932_MOESM1_ESM.pdf]

Additional file 1

A1. Deterministic model and equilibria

The deterministic equations in the presence of mutations and their equilibrium solutions are shown below for the resistant X chromosome (Model I) and the trans-acting suppressor (Model II).

A1.1. Model I

Populations of genotypes with no resistant X chromosomes are denoted as $H(t), M(t), F(t)$, heterozygotes with one resistant X chromosome as $H_R(t), M_R(t), F_R(t)$, and homozygous females with two resistant X chromosomes as $F_{RR}(t)$. H refers to males with the driving Y chromosome, and M to males with the wild-type Y chromosome; the driving Y is transmitted to a proportion m of a male's progeny. We define u $(0 \leq u \leq 1)$ as the fraction of female progeny of a driving Y male that inherit a resistant X chromosome. While it is assumed that the presence of the driving Y chromosome does not affect fitness other than the sex ratio bias, males and females with the resistant X may suffer fitness costs that are manifest as differences in their relative ability to participate in mating and reproduction. More precisely, for fitness $w_i \leq 1$ of mutant type Z_i with the resistant X (relative to fitness one for non-mutants), effectively only a reduced number of such individuals, given by $w_i\, Z_i$, participate in reproduction and produce gametes (all types are assumed to contribute equally to the density-dependent recruitment rate $[R_m - \gamma N(t)]$) [1]. The rate of change of the total population size $N(t) = H(t) + M(t) + F(t) + H_R(t) + M_R(t) + F_R(t) + F_{RR}(t)$ (i.e., the total number of individuals) is therefore given by:

$$\frac{dN(t)}{dt} = 2[R_m - \gamma N(t)]\left(F(t) + w_{F_R}F_R(t) + w_{F_{RR}}F_{RR}(t)\right) - N(t)$$

$$= 2[R_m - \gamma N(t)]\overline{w}_f(t)N_f(t) - N(t) \qquad \text{(A1.1)}$$

which is also written in terms of the average relative female fitness, $\overline{w}_f(t) = \frac{F(t)+w_{F_R}F_R(t)+w_{F_{RR}}F_{RR}(t)}{N_f(t)}$, where the total female population is $N_f(t) = F(t) + F_R(t) + F_{RR}(t)$. Thus the total recruitment rate is dependent on the number and fitness of females, and the assumption that all participating females are fertilized [2]. Time is normalized with generation time, since the equations then depend on only one growth parameter, $R_m = \lambda/\mu$ (the intrinsic growth rate of the population) rather than on two (the density-independent birth rate λ and death rate μ).

The equations for the individual genotype populations are now considered, following a previous model [1], but here differentiating between males and females. It is assumed that individuals that participate in mating choose partners at random and, for simplicity, assume that each offspring is from an independent mating event, such that the genotypic composition of offspring is equal to draws of pairs of gametes from the available 'pool'. For example, among all offspring produced, the fraction of mutants $H_R\,(t)$ (driving Y males with the resistant X) is given by the proportion of gametes contributed by participating females with a resistant X chromosome, $g_{[f,X^R]}(t)$, and the proportion of gametes contributed by participating males with a driving Y chromosome, $g_{[a,Y^D]}(t)$:

$$g_{[f,X^R]}(t)g_{[a,Y^D]}(t) = \left(\frac{w_{F_R}F_R(t)/2 + w_{F_{RR}}F_{RR}(t)}{\overline{w}_f(t)N_f(t)}\right)\left(\frac{mH(t) + w_{H_R}H_R(t)/2}{\overline{w}_a(t)N_a(t)}\right)$$

Above, $\overline{w}_a N_a(t)$ is the total number of males participating in mating, with average relative male fitness defined as $\overline{w}_a(t) = \frac{H(t)+M(t)+w_{H_R}H_R(t)+w_{M_R}M_R(t)}{N_a(t)}$ and total male population given by $N_a(t) = H(t) + M(t) + H_R(t) + M_R(t)$. The total rate of production of new offspring, $2[R_m - \gamma N(t)]\overline{w}_f(t)N_f(t)$, is then multiplied by $g_{[f,X^R]}(t)g_{[a,Y^D]}(t)$ to obtain the recruitment rate and the equation for the rate of change of $H_R(t)$:

$$\frac{dH_R(t)}{dt} = 2[R_m - \gamma N(t)]\overline{w}_f(t)N_f(t)g_{[f,X^R]}(t)g_{[a,Y^D]}(t) \; - H_R(t)$$

Similar equations are constructed for all other genotypes, with the fraction of offspring $F_R(t)$ calculated as the fraction of births for which the resistant X is contributed by the female and the wild-type X by the males, and vice versa: $g_{[f,X^R]}(t)g_{[a,X]}(t) + g_{[f,X]}(t)g_{[a,X^R]}(t)$, taking into account the fraction u of Xs from driving Y males that are resistant. Selection therefore occurs through the relative participation of different genotypes in mating and reproduction. Since total recruitment rates are dependent on the female population (A1.1), fitness cost to the resistant X therefore causes an effective reduction in fertility for females, and reduced participation in mating for males, as given by the resulting nonlinear system of seven differential equations:

$$\frac{dH(t)}{dt} = 2[R_m - \gamma N(t)]\left(F(t) + \frac{w_{F_R}F_R(t)}{2}\right)\left(\frac{2mH(t)+w_{H_R}H_R(t)}{2\overline{w}_a(t)N_a(t)}\right) - H(t)$$

$$\frac{dM(t)}{dt} = 2[R_m - \gamma N(t)]\left(F(t) + \frac{w_{F_R}F_R(t)}{2}\right)\left(\frac{M(t)+w_{M_R}M_R(t)}{2\overline{w}_a(t)N_a(t)}\right) - M(t)$$

$$\frac{dF(t)}{dt} = 2[R_m - \gamma N(t)]\left(F(t) + \frac{w_{F_R}F_R(t)}{2}\right)\left(\frac{M(t)+2(1-u)(1-m)H(t)}{2\overline{w}_a(t)N_a(t)}\right) - F(t)$$

$$\frac{dH_R(t)}{dt} = 2[R_m - \gamma N(t)]\left(\frac{w_{F_R}F_R(t)}{2} + w_{F_{RR}}F_{RR}(t)\right)\left(\frac{2mH(t)+w_{H_R}H_R(t)}{2\overline{w}_a(t)N_a(t)}\right) - H_R(t)$$

$$\frac{dM_R(t)}{dt} = 2[R_m - \gamma N(t)]\left(\frac{w_{F_R}F_R(t)}{2} + w_{F_{RR}}F_{RR}(t)\right)\left(\frac{M(t)+w_{M_R}M_R(t)}{2\overline{w}_a(t)N_a(t)}\right) - M_R(t)$$

$$\frac{dF_R(t)}{dt} = 2[R_m - \gamma N(t)]\left[\left(F(t) + \frac{w_{F_R}F_R(t)}{2}\right)\left(\frac{w_{M_R}M_R(t)+w_{H_R}H_R(t)+2u(1-m)H(t)}{2\overline{w}_a(t)N_a(t)}\right) + \left(\frac{w_{F_R}F_R(t)}{2} + w_{F_{RR}}F_{RR}(t)\right)\left(\frac{M(t)+2(1-u)(1-m)H(t)}{2\overline{w}_a(t)N_a(t)}\right)\right] - F_R(t)$$

$$\frac{dF_{RR}(t)}{dt} = 2[R_m - \gamma N(t)]\left(\frac{w_{F_R}F_R(t)}{2} + w_{F_{RR}}F_{RR}(t)\right)\left(\frac{w_{M_R}M_R(t)+w_{H_R}H_R(t)+2u(1-m)H(t)}{2\overline{w}_a(t)N_a(t)}\right) - F_{RR}(t)$$

(A1.2)

Driving Y males are introduced at $t = 0$ into a wild-type mosquito population of $N_0 = \frac{R_m - 1}{\gamma}$, and an exemplar solution for baseline parameters and no fitness cost is shown in Fig. A1.1.

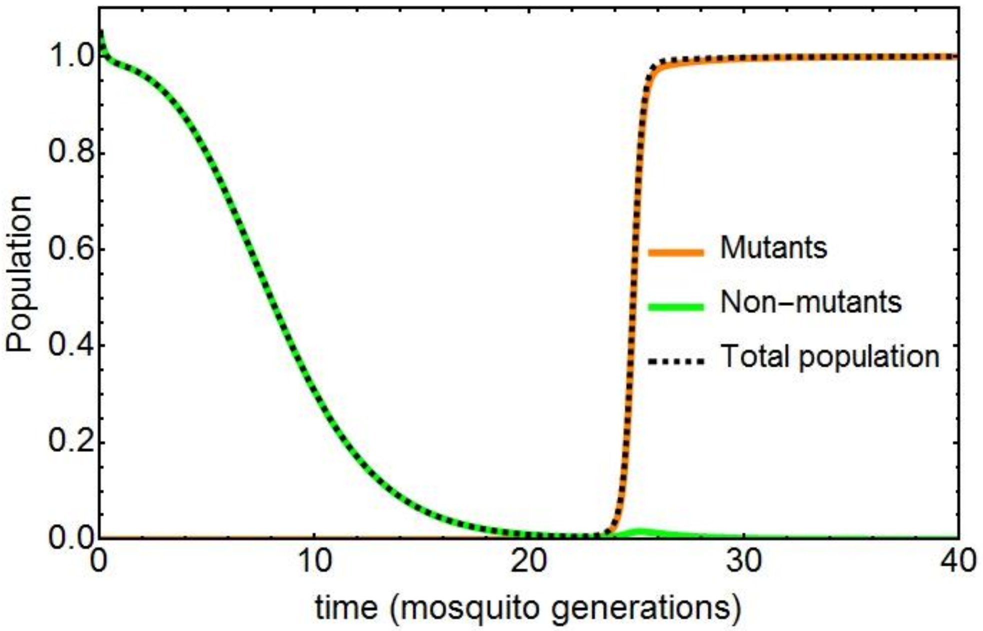

Figure A1.1. Example time course for the deterministic model with mutation ($u = 10^{-6}$) for cost-free resistance, showing initial spread of the driving Y after its release at $t = 0$, followed by spread of the resistant X, restoring a 50:50 sex ratio. Populations are normalized by the wild-type pre-release population N_0, and parameter values are $R_m = 6, m = 0.95, h_0 = 0.05, \gamma = (R_m - 1)/N_0$.

A1.1a Seasonal variation

Periodic variation is modelled with a sinusoidal time dependence in the density-dependent part of the mosquito recruitment rate, $\gamma(t) = \gamma_0[a]\left(1 + a\sin\left[\frac{2\pi t}{T}\right]\right)$. T is the period of the seasonality, and a is the amplitude of the oscillations. To avoid a negative recruitment rate, which may occur for large seasonal variations in the population, it is specified that the total seasonal recruitment rate $\Lambda(t)$ cannot go below zero:

$$\Lambda(t) = \text{Max}[0, R_m - \gamma_0[a]\left(1 + a\sin\left[\frac{2\pi t}{T}\right]\right) N(t)]$$

To find the pre-release wild-type equilibrium, the deterministic equations are solved numerically for the wild-type population [(1) in the main article], with non-negative recruitment rate given by $\Lambda(t)$, until the solution has converged to its long-time periodic equilibrium $N_{eq}(a,t)$. The average wild-type population size over a period is $\bar{N}(a) = \frac{1}{T}\lim_{s\to\infty}\int_s^{s+T} N_{eq}(a,t)\,dt$. It is an (increasing) function of the amplitude a, so the density-dependent mortality constant is set to $\gamma_0[a] = \gamma_0\frac{\bar{N}(a)}{N_0}$ to maintain a constant average population of wild-type mosquitoes over a period for all amplitudes.

A1.1b Equilibria

To determine the equilibrium populations in the presence of mutations for the case of no seasonal variation, the time derivatives in (A1.2) are set equal to zero to yield nonlinear algebraic equations for

the time-independent population sizes. Using Wolfram Mathematica [3] to aid us with algebraic manipulation and simplification of large expressions, analytical solutions are derived for the individual populations $H, M, F, H_R, M_R, F_R,$ and F_{RR} (and thus total population N) as functions of R_m, m, u and mutant fitnesses $w_{F_R}, w_{H_R}, w_{M_R}, w_{F_{RR}}$. For simplicity, it is assumed that $w_{F_R} = w$, $w_{H_R} = w_{M_R} = w_{F_{RR}} = w^2$.

In the equilibria below, the populations are normalized with N_0, and $\gamma = (R_m - 1)/N_0$ is used.

Fixation/intermediate equilibrium. When the solutions are expressed as fractions of individual types in the total population (e.g. $f = F/N$), it is found that for fitness $w \geq w_1[m, u]$ (independent of R_m), the proportions of non-mutants and heterozygote females in the total population are zero. Therefore, w_1 is the demarcation between the region where resistant mutations are fixed in the population ($w_1 \leq w \leq 1$) and the region of intermediate equilibrium ($0 < w < w_1$). We derive w_1 by setting the solutions for the proportions of non-mutants and heterozygote females to zero to obtain:

$$w_1 = \frac{1}{6}\left(1 + A + \frac{1}{A}\right)$$

where

$$A = \sqrt[3]{1 + 108(1-m)(1-u) + 2\sqrt{54(1-m)(1-u)(1 + 54(1-m)(1-u))}}$$

Population suppression/extinction. The region where the total population is eliminated is now considered. We start with the equation for the total population (A1.1) at equilibrium, which includes all the female populations. It is more convenient to work with populations normalized with N_0, e.g. $\widetilde{N}(t) = \frac{N(t)}{N_0}$, and it is found that N_0 cancels out and vanishes in (A1.1) except inside the density-dependent recruitment rate:

$$R_m - \gamma N(t) = R_m - \gamma N_0 \left(\frac{N(t)}{N_0}\right) = R_m - (\gamma N_0)\widetilde{N}(t).$$

If $\gamma N_0 = (R_m - 1)$ is substituted above, one obtains the recruitment rate in terms of normalized population: $R_m - \gamma N(t) \rightarrow R_m - (R_m - 1)\widetilde{N}(t)$. Henceforth, for the equations in this section, instead of referring to all normalized populations with a tilde sign (e.g. $\widetilde{N}(t)$), which would be cumbersome, it is specified that all populations N, F below refer to populations normalized by N_0. We obtain from (A1.1) for normalized populations:

$$2[R_m - (R_m - 1)N](F + wF_R + w^2 F_{RR}) = N$$

Since substituting in zero for all the populations in this equation does not yield a solution, we rewrite it in terms of the population frequencies, e.g. $f_R = \frac{F_R}{N}, f_{RR} = \frac{F_{RR}}{N}$:

$$\frac{2R_m(f + wf_R + w^2 f_{RR}) - 1}{2(f + wf_R + w^2 f_{RR})(R_m - 1)} = N$$

We then set $N = 0$ above, which gives the condition for zero total population:

$$2R_m(f + wf_R + w^2 f_{RR}) = 1 \tag{A1.3}$$

For $w_1 \le w \le 1$, where the mutation is fixed, the total population is zero to the left of a line that is defined as the 'extinction' line, $w = w_{\mathrm{ex}}[R_m]$ (see Fig. 3 in main article). To the right of w_{ex}, the population is non-zero and with the mutation fixed in the population (composed 50:50 of females and males and with H, M, F, F_R equal to zero):

$$N = \frac{w^2 R_m - 1}{w^2(R_m - 1)} \tag{A1.4}$$

To obtain the fitness below which there is 100% suppression of the population, $f_{RR} = 1/2$ and $f, f_R = 0$ are substituted into (A1.3) to obtain $w_{\mathrm{ex}} = 1/\sqrt{R_m}$ for $w_1 \le w \le 1$ (equivalently, one can substitute $N = 0$ in (A1.4) and solve for $w = w_{\mathrm{ex}}$).

For $0 < w < w_1$, at intermediate equilibrium, the population is zero below the line $w = w_{\mathrm{ex}}[R_m, m, u]$ and there is a reduced non-zero population above the line (Fig. 3 in main article). The analytical solutions that are derived for the populations for intermediate equilibrium are not shown here due to length and complexity. We substitute in the expressions for the fractions of female populations in (A1.3) and solve for w_{ex}; we obtain the following expression relating R_m and w_{ex} for $0 < w < w_1$:

$$R_m = \frac{B_1[w_{\mathrm{ex}}, m, u]}{B_2[w_{\mathrm{ex}}, m, u]} \tag{A1.5}$$

where

$$B_1 = 2(1 - w_{\mathrm{ex}})\Big((w_{\mathrm{ex}} - 3)w_{\mathrm{ex}}^3 - 2(1-m)\big(2 - w_{\mathrm{ex}} + w_{\mathrm{ex}}^2 + 2u(-1 + w_{\mathrm{ex}} - w_{\mathrm{ex}}^2 + w_{\mathrm{ex}}^3)\big) - (1 + w_{\mathrm{ex}})\, B_3\Big),$$

$$B_2 = w_{\mathrm{ex}}^6 - 4(1-m)w_{\mathrm{ex}}^3\big(-2 + 3w_{\mathrm{ex}} + u(2 - 3w_{\mathrm{ex}} + w_{\mathrm{ex}}^2)\big) - 4(1-m)^2\big(4 + 4u^2(-1 + w_{\mathrm{ex}})^2 - 4w_{\mathrm{ex}} - w_{\mathrm{ex}}^2 - 2u(4 - 6w_{\mathrm{ex}} + w_{\mathrm{ex}}^2 + w_{\mathrm{ex}}^3)\big) + \big(w_{\mathrm{ex}}^3 + (1-m)(-4 + 4u + 2w_{\mathrm{ex}} - 4uw_{\mathrm{ex}})\big)\, B_3,$$

and

$$B_3 = \big[16(1-m)^2u^2(1 - w_{\mathrm{ex}})^2(1 + w_{\mathrm{ex}}^2) + (2(1-m)(-2 + w_{\mathrm{ex}}) + w_{\mathrm{ex}}^3)^2 + 8(1-m)u(w_{\mathrm{ex}} - 1)\big(w_{\mathrm{ex}}^3(1 + w_{\mathrm{ex}}) - 2(1-m)(2 - w_{\mathrm{ex}} + w_{\mathrm{ex}}^2)\big)\big]^{\frac{1}{2}}$$

For a given R_m, this could be solved numerically for w_{ex}; in practice for plotting (Fig. 3 in main article), it is easier to calculate R_m for a given $w_{\mathrm{ex}} < w_1$.

Fig. A1.2 shows the calculated line w_{ex} as a function of R_m for a range of values of m (transmission rate of driving Y); below and to the left of each line, there is extinction of the population.

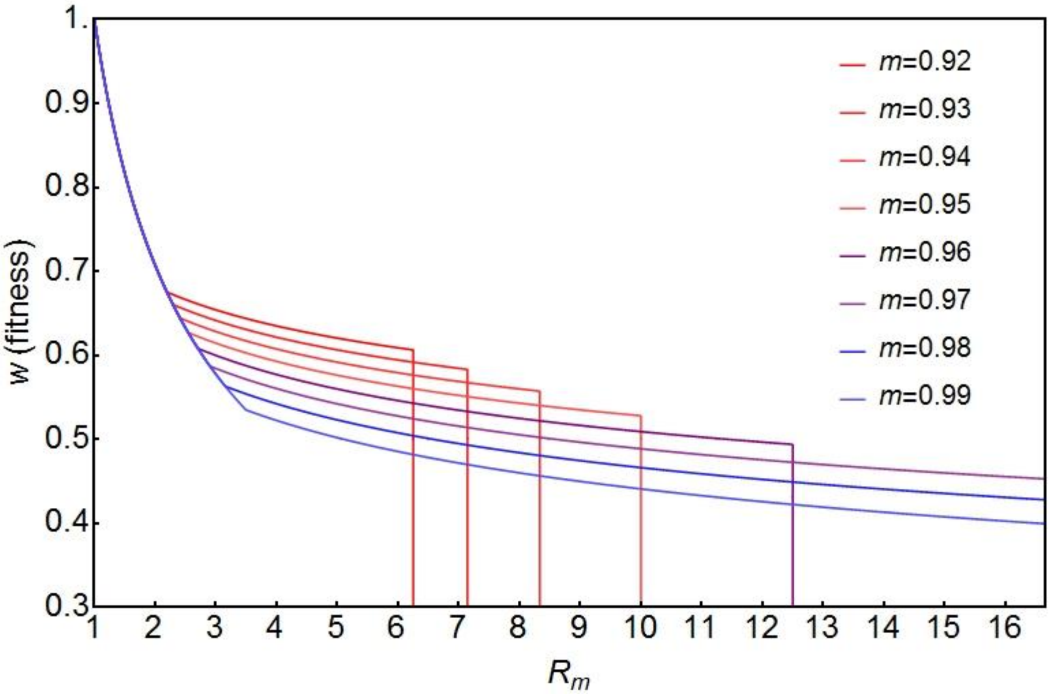

Figure A1.2. Outcome of equilibria for resistant X chromosome mutation (Model I) for a range of values of m (transmission rate of the driving Y). The lines represent $w = w_{ex}$ (100% population suppression) as a function of the fitness parameter w (for heterozygous females, with fitness w^2 for homozygous females and hemizygous males) and the intrinsic rate of increase of the population (R_m). Here, $u = 10^{-6}$. Below and to the left of $w = w_{ex}$ for each m, the total population is zero; above and to the right, the population is nonzero. For each m, the population is always nonzero for $R_m \geq \frac{1}{2(1-m)(1-u)}$, where the Y drive is not sufficient to eliminate the population.

In addition to the equation for w_{ex}, we also calculate the lines $w = w_\varphi\ [\varphi,\ R_m, m, u]$ that represent the values for which the total female population is a fraction φ of its value of $\frac{1}{2}$ when there is no fitness cost:

$$F + F_R + F_{RR} = \frac{\varphi}{2} \quad \text{(A1.6)}$$

Note that $w_{\varphi=0} = w_{\text{ex}}$. For $w_1 \leq w \leq 1$, where the mutation is fixed in the population, the total female population F_{RR} is half of the total population N (A1.4) and substituting F_{RR} into (A1.6) above yields:

$$\frac{\varphi}{2} = \frac{w^2 R_m - 1}{2w^2(R_m - 1)} \quad \text{(A1.7)}$$

This can be rewritten to give $w_\varphi = 1/\sqrt{R_m - \varphi(R_m - 1)}$.

Below w_1, in the region of intermediate equilibrium, one may substitute the analytical expressions for the female populations for intermediate equilibrium into (A1.6) and rearrange to obtain:

$$R_m = B_4[w_\varphi, m, u, \varphi]$$

where B_4 is too complex to show here (and reduces to B_1/B_2 for $\varphi = 0$). Since this equation yields R_m for a given w_φ, it is used to construct Fig. 3 in the main paper. Each constant female population line $w = w_\varphi$ intersects the line $w = w_1$ (that separates the total fixation and intermediate equilibrium regions) at $R_m = R_m^*(\varphi)$. We obtain R_m^* by substituting w_1 in (A1.7) above:

$$R_m^* = \frac{1 - w_1^2\,\varphi}{w_1^2(1-\varphi)}$$

To find the region where the total population persists for all fitnesses, one can substitute $w = 0$ in the solution for w_{ex} for $0 \le w < w_1$ (A1.5) to obtain:

$$R > R_{m,crit} = \frac{1}{2(1-m)(1-u)}$$

A1.2 Model II

For this model, populations of heterozygotes with one trans-acting suppressor allele are denoted by $F_S(t), H_S(t), M_S(t)$ and homozygotes by $F_{SS}(t), H_{SS}(t), M_{SS}(t)$. Of all births (male or female), the chance of the suppressor mutation arising on the relevant autosome is v ($0 \le v \le 1$) per individual per autosome. For example, for births from parents with only non-mutant alleles, a fraction $2v$ of their offspring will have a suppressor mutation, since either autosome in the offspring could mutate independently (the probability of v^2 of two mutations occurring at the same time is ignored, since the focus is on $v \ll 1$). For costly resistance, each mutant type with one or two suppressor alleles has a decreased fitness of $w_{Fs}, w_{Hs}, w_{Ms}, w_{Fss}, w_{Hss}, w_{Mss} \le 1$. The same approach as for Model I above is used, except that gametes with or without the suppressor allele that are contributed by males are further differentiated by whether they also have an X, Y or driving Y chromosome and therefore result in female, wild-type male or driving Y male progeny. The time-dependent ODEs for the mutant and non-mutant populations are (with an exemplar solution for baseline parameters and no fitness cost in Fig. A1.3):

$$\frac{dH(t)}{dt} = 2[R_m - \gamma N(t)](1-2v)\left(F(t) + \frac{w_{F_S}F_S(t)}{2}\right)\left(\frac{4mH(t) + w_{H_S}H_S(t)}{4\bar{w}_a(t)N_a(t)}\right) - H(t)$$

$$\frac{dM(t)}{dt} = 2[R_m - \gamma N(t)](1-2v)\left(F(t) + \frac{w_{F_S}F_S(t)}{2}\right)\left(\frac{2M(t) + w_{M_S}M_S(t)}{4\bar{w}_a(t)N_a(t)}\right) - M(t)$$

$$\frac{dF(t)}{dt} = 2[R_m - \gamma N(t)](1-2v)\left(F(t) + \frac{w_{F_S}F_S(t)}{2}\right)\left(\frac{2M(t) + 4(1-m)H(t) + w_{M_S}M_S(t) + w_{H_S}H_S(t)}{4\bar{w}_a(t)N_a(t)}\right) - F(t)$$

$$\frac{dH_S(t)}{dt} = 2[R_m - \gamma N(t)]\left[F(t)\left(\frac{8mv\,H(t) + (1+v)w_{H_S}H_S(t) + 2(1-v)w_{H_{SS}}H_{SS}(t)}{4\bar{w}_a(t)N_a(t)}\right) + w_{F_S}F_S(t)\left(\frac{2m(1+v)H(t) + w_{H_S}H_S(t) + (1-v)w_{H_{SS}}H_{SS}(t)}{4\bar{w}_a(t)N_a(t)}\right) + w_{F_{SS}}(1-v)F_{SS}(t)\left(\frac{4mH(t) + w_{H_S}H_S(t)}{4\bar{w}_a(t)N_a(t)}\right)\right] - H_S(t)$$

$$\frac{dM_S(t)}{dt} = 2[R_m - \gamma N(t)]\left[F(t)\left(\frac{4v\,M(t) + (1+v)w_{M_S}M_S(t) + 2(1-v)w_{M_{SS}}M_{SS}(t)}{4\bar{w}_a(t)N_a(t)}\right) + w_{F_S}F_S(t)\left(\frac{(1+v)M(t) + w_{M_S}M_S(t) + (1-v)w_{M_{SS}}M_{SS}(t)}{4\bar{w}_a(t)N_a(t)}\right) + w_{F_{SS}}(1-v)F_{SS}(t)\left(\frac{2M(t) + w_{M_S}M_S(t)}{4\bar{w}_a(t)N_a(t)}\right)\right] - M_S(t)$$

$$\frac{dF_S(t)}{dt} = 2[R_m - \gamma N(t)] \left[F(t)\left(\frac{vM(t)+2v(1-m)H(t)}{\bar{w}_a(t)N_a(t)} + \frac{(1+v)(w_{M_S}M_S(t)+w_{H_S}H_S(t))}{4\bar{w}_a(t)N_a(t)} + \frac{(1-v)(w_{M_{SS}}M_{SS}(t)+w_{H_{SS}}H_{SS}(t))}{2\bar{w}_a(t)N_a(t)}\right) + w_{F_S}F_S(t)\left(\frac{(1+v)(M(t)-2(1-m)H(t))}{4\bar{w}_a(t)N_a(t)} + \frac{w_{M_S}M_S(t)+w_{H_S}H_S(t)}{4\bar{w}_a(t)N_a(t)} + \frac{(1-v)(w_{M_{SS}}M_{SS}(t)+w_{H_{SS}}H_{SS}(t))}{4\bar{w}_a(t)N_a(t)}\right) + (1-v)w_{F_{SS}}F_{SS}(t)\left(\frac{M(t)+2(1-m)H(t)}{2\bar{w}_a(t)N_a(t)} + \frac{w_{M_S}M_S(t)+w_{H_S}H_S(t)}{4\bar{w}_a(t)N_a(t)}\right)\right] - F_S(t)$$

$$\frac{dH_{SS}(t)}{dt} = 2[R_m - \gamma N(t)] \left[F(t)\left(\frac{v\,w_{H_S}H_S(t)+2v\,w_{H_{SS}}H_{SS}(t)}{4\bar{w}_a(t)N_a(t)}\right) + w_{F_S}F_S(t)\left(\frac{4v\,mH(t)+(1+2v)w_{H_S}H_S(t)+2(1+v)w_{H_{SS}}H_{SS}(t)}{8\bar{w}_a(t)N_a(t)}\right) + w_{F_{SS}}F_{SS}(t)\left(\frac{4v\,mH(t)+(1+v)w_{H_S}H_S(t)+2w_{H_{SS}}H_{SS}(t)}{4\bar{w}_a(t)N_a(t)}\right)\right] - H_{SS}(t)$$

$$\frac{dM_{SS}(t)}{dt} = 2[R_m - \gamma N(t)] \left[F(t)\left(\frac{v\,w_{M_S}M_S(t)+2v\,w_{M_{SS}}M_{SS}(t)}{4\bar{w}_a(t)N_a(t)}\right) + w_{F_S}F_S(t)\left(\frac{2v\,M(t)+(1+2v)w_{M_S}M_S(t)+2(1+v)w_{M_{SS}}M_{SS}(t)}{8\bar{w}_a(t)N_a(t)}\right) + w_{F_{SS}}F_{SS}(t)\left(\frac{2v\,M(t)+(1+v)w_{M_S}M_S(t)+2w_{M_{SS}}M_{SS}(t)}{4\bar{w}_a(t)N_a(t)}\right)\right] - M_{SS}(t)$$

$$\frac{dF_{SS}(t)}{dt} = 2[R_m - \gamma N(t)] \left[vF(t)\left(\frac{w_{M_S}M_S(t)+w_{H_S}H_S(t)}{4\bar{w}_a(t)N_a(t)} + \frac{w_{M_{SS}}M_{SS}(t)+ w_{H_{SS}}H_{SS}(t)}{2\bar{w}_a(t)N_a(t)}\right) + w_{F_S}F_S(t)\left(\frac{vM(t)+2v(1-m)H(t)}{4\bar{w}_a(t)N_a(t)} + \frac{(1+2v)(w_{M_S}M_S(t)+w_{H_S}H_S(t))}{8\bar{w}_a(t)N_a(t)} + \frac{(1+v)(w_{M_{SS}}M_{SS}(t)+w_{H_{SS}}H_{SS}(t))}{4\bar{w}_a(t)N_a(t)}\right) + w_{F_{SS}}F_{SS}(t)\left(\frac{v(M(t)+2(1-m)H(t))}{2\bar{w}_a(t)N_a(t)} + \frac{(1+v)(w_{M_S}M_S(t)+w_{H_S}H_S(t))}{4\bar{w}_a(t)N_a(t)} + \frac{w_{M_{SS}}M_{SS}(t)+w_{H_{SS}}H_{SS}(t)}{2\bar{w}_a(t)N_a(t)}\right)\right] - F_{SS}(t)$$

(A1.8)

Above, the total number of individuals is $N(t) = H(t) + M(t) + F(t) + H_S(t) + M_S(t) + F_S(t) + H_{SS}(t) + M_{SS}(t) + F_{SS}(t)$. $\bar{w}_a N_a(t)$ is the total number of males participating in mating, with average relative male fitness defined as $\bar{w}_a(t) = \frac{H(t)+M(t)+w_{H_S}H_S(t)+w_{M_S}M_S(t)+w_{H_{SS}}H_{SS}(t)+w_{M_{SS}}M_{SS}(t)}{N_a(t)}$ and total male population given by $N_a(t) = H(t) + M(t) + H_S(t) + M_S(t) + H_{SS}(t) + M_{SS}(t)$. The differential equation for the total population is given by:

$$\frac{dN(t)}{dt} = 2[R_m - \gamma N(t)]\left(F(t) + w_{F_S}F_S(t) + w_{F_{SS}}F_{SS}(t)\right) - N(t)$$

$$= 2[R_m - \gamma N(t)]\bar{w}_f N_f(t) - N(t) \qquad \text{(A1.9)}$$

where, as before, $\bar{w}_f$ is the average relative female fitness and $N_f(t)$ is the total number of females.

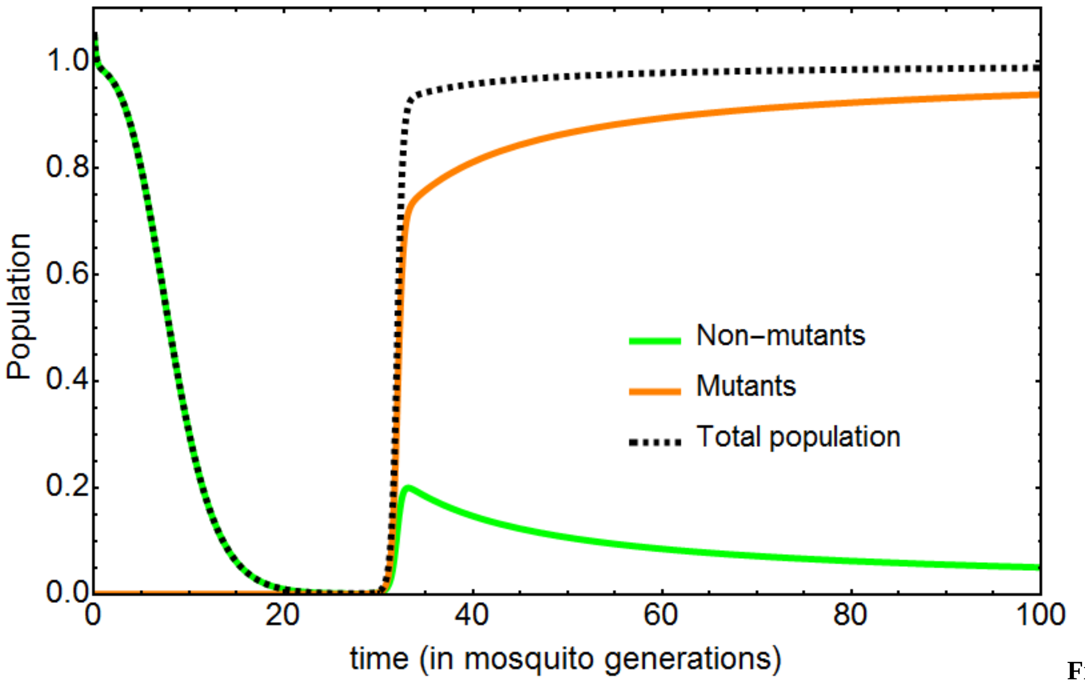

Figure A1.3. Example time course for the deterministic model with mutation ($v = 10^{-7}$) for cost-free resistance, showing initial spread of the driving Y after its release at $t = 0$, followed by spread of the suppressor mutation, restoring a 50:50 sex ratio. Populations are normalized with the wild-type pre-release population N_0, and parameter values are $R_m = 6, m = 0.95, h_0 = 0.05, \gamma = (R_m - 1)/N_0$.

A1.2a Equilibria

For the equilibria for the trans-acting suppressor mutation on an autosome, as with Model I, the time derivatives in (A1.8) are set equal to zero to yield nonlinear algebraic equations for the population sizes. Due to the greater complexity of the model (nine types), we are unable to obtain analytical solutions for the individual populations $H, M, F, H_S, M_S, F_S, H_{SS}, M_{SS}, F_{SS}$ (and thus total population N) as functions of R_m, m, v and mutant fitnesses $w_{F_S}, w_{H_S}, w_{M_S}, w_{F_{SS}}, w_{H_{SS}}, w_{M_{SS}}$, even with the simplifications $w_{F_S} = w_{H_S} = w_{M_S} = w$ and $w_{F_{SS}} = w_{H_{SS}} = w_{M_{SS}} = w^2$.

In the equilibria below, the populations are normalized with N_0, and $\gamma = \frac{R_m - 1}{N_0}$ is used.

Fixation/intermediate equilibrium. As with Model I, there is a fitness $w \geq w_1[m, v]$ (independent of R_m), that separates the region where resistant mutations are fixed in the population ($w_1 \leq w \leq 1$) and the region of intermediate equilibrium ($0 < w < w_1$). Although we are not able to obtain analytical solutions for each population in the intermediate equilibrium region, the nine equilibrium equations are reduced to two equations for the fitness-weighted proportions of males and of females (not shown due to complexity). w_1 is found by setting these two quantities to their fixation region value of $w^2/2$ (i.e., population of non-mutants and heterozygotes to zero and of homozygotes to ½) to obtain:

$$w_1 = 1 - v$$

Population suppression/extinction. The focus is now on the two regions of zero and non-zero population. To obtain w_{ex}, one starts with the long-time limit of equation (A1.8) for the total population, which includes all the female populations:

$$2[R_m - (R_m - 1)N](F + wF_S + w^2F_{SS}) = N$$

This can be rewritten in terms of the proportions, e.g. $= \frac{F}{N}, f_S = \frac{F_S}{N}, f_{SS} = \frac{F_{SS}}{N}$; by solving for N and then setting $N = 0$, the condition for zero total population is obtained:

$$2R_m(f + wf_S + w^2f_{SS}) = 1 \quad \text{(A1.10)}$$

For $w_1 \leq w \leq 1$, where the mutation is fixed, the total population is zero below the extinction line $w = w_{\text{ex}}[R_m]$ (Fig. 7, main article). Above w_{ex}, the population is non-zero and with the mutation fixed in the population (composed 50:50 of homozygote females and males and with non-mutants and heterozygotes H, M, F, H_S, M_S, F_S equal to zero):

$$N = \frac{w^2R_m - 1}{w^2(R_m - 1)} \quad \text{(A1.11)}$$

As in Model I, it is found that $w_{\text{ex}} = 1/\sqrt{R_m}$ for $1 - v \leq w \leq 1$.

For $0 < w < w_1$, at intermediate equilibrium, the condition for zero population is given by (A1.10). For this more complex case, analytical solutions for the female proportions were unobtainable, so $w_{\text{ex}}[R_m, m, v]$ is calculated using a different approach. The full set of equilibrium equations for the individual populations is solved numerically using Wolfram Mathematica FindRoot [3] for a given w, R_m, m, v. An iterative numerical method is then used to find $w = w_{\text{ex}}$, the maximum fitness for which the population is eliminated (Fig. 7, main paper).

We also determine the lines $w = w_\varphi\ [\varphi,\ R_m, m, v]$ for which the total female population is a fraction φ of its value of $\frac{1}{2}$ when there is no fitness cost:

$$F + F_S + F_{SS} = \frac{\varphi}{2} \quad \text{(A1.12)}$$

For $w_1 \leq w \leq 1$, where the mutation is fixed in the population, the total female population F_{SS} is half of the total population N (A1.11), and substituting F_{SS} into (A1.12) above yields, as for Model I:

$$w_\varphi = 1/\sqrt{R_m - \varphi(R_m - 1)}$$

Below w_1, in the region of intermediate equilibrium, the equilibrium equations for the individual populations are solved numerically using Wolfram Mathematica FindRoot for a given w, R_m, m, v. As above for w_{ex}, we iterate to find $w = w_\varphi$ where the total female population is $\varphi/2$ (Fig. 7, main paper).

A2. Branching process method

The time-inhomogeneous branching process method is used to calculate probabilities of stochastic loss. It is assumed in this model that stochastic loss of a new mutation occurs in the early stages when the mutant population size is extremely small (i.e., once the mutation has survived this early phase, it will

establish with high probability), and therefore the numbers of individuals without the resistant mutation are much greater than the numbers of non-mutants (see [4]). As is standard, we therefore treat the non-mutant populations, $F(t), M(t), H(t)$, deterministically using (2) in the main article, and treat the mutants with the resistant gene stochastically via a branching process model. For the calculation of the probabilities of stochastic loss, one may then ignore second order terms and homozygous individuals $F_{RR}(t)$.

Henceforth, all non-mutant populations are normalized with N_0, and $\gamma = (R_m - 1)/N_0$ is used.

A2.1 Model I

By applying the linearization assumptions above in the equations for heterozygotes with the resistant gene (A1.2), one may construct a branching process for the resistant types based on birth and death rates:

$$H_R \rightarrow H_R + 1 \qquad \frac{\big(R_m-(R_m-1)N(t)\big)mH(t)}{A(t)} w_{F_R} F_R$$

$$M_R \rightarrow M_R + 1 \qquad \frac{\big(R_m-(R_m-1)N(t)\big)M(t)}{2A(t)} w_{F_R} F_R \tag{A2.1}$$

$$F_R \rightarrow F_R + 1 \qquad \frac{2uN_0(1-m)\big(R_m-(R_m-1)N(t)\big)F(t)H(t)}{A(t)} + \frac{\big(R_m-(R_m-1)N(t)\big)}{A(t)}\Big(F(t)(w_{M_R}M_R + w_{H_R}H_R) + w_{F_R}F_R(M(t)/2 + (1-m)H(t))\Big)$$

$$H_R \rightarrow H_R - 1 \qquad H_R$$

$$M_R \rightarrow M_R - 1 \qquad M_R$$

$$F_R \rightarrow F_R - 1 \qquad F_R$$

Above, $N(t) = H(t) + M(t) + F(t)$ and $A(t) = H(t) + M(t)$. $H(t), M(t), F(t)$ are solved numerically from the deterministic equation (2) for non-mutants in the main article, with time zero corresponding to introduction of the driving Y in a carrying capacity landscape of wild types. The first term in the expression for the birth term for $F_R(t)$ above is the population-wide rate at which female mutants first arise from mutation,

$$r_{Mut}(t) = \frac{2uN_0(1-m)\big(R_m-(R_m-1)N(t)\big)F(t)H(t)}{A(t)} \tag{A2.2}$$

Note that if the fitnesses of mutant males with and without the driving Y are the same, $w_{H_R} = w_{M_R} = w_{A_R}$, one may then introduce $A_R(t) = H_R(t) + M_R(t)$, and (A2.1) reduces to:

$$A_R \rightarrow A_R + 1 \qquad \frac{\big(R_m-(R_m-1)N(t)\big)(2mH(t)+M(t))}{2A(t)} w_{F_R} F_R \tag{A2.3}$$

$F_R \rightarrow F_R + 1$ $\quad \frac{2uN_0(1-m)\left(R_m-(R_m-1)N(t)\right)F(t)H(t)}{A(t)} + \frac{R_m-(R_m-1)N(t)}{A(t)}\left(F(t)\, w_{A_R}A_R + w_{F_R}F_R(M(t)/2 + (1-m)H(t))\right)$

$A_R \rightarrow A_R - 1$ $\quad A_R$

$F_R \rightarrow F_R - 1$ $\quad F_R$

Returning to the general case (A2.1), $p_{i,j,k}(t)$ is denoted as the probability of i, j, k mutant individuals of type H_R, M_R, F_R at time t. The Kolmogorov forward equation is written for the temporal evolution of the probabilities $p_{i,j,k}(t)$ according to the birth-death processes in (A2.1):

$$\frac{p_{i,j,k}(t+\Delta t)}{\Delta t} = p_{i,j,k}(t) + \left[(i+1)p_{i+1,j,k}(t) - i\,p_{i,j,k}(t) + (j+1)p_{i,j+1,k}(t) - j\,p_{i,j,k}(t) + (k+1)\,p_{i,j,k+1}(t) - k\,p_{i,j,k}(t)\right]$$

$$+ \frac{\left(R_m-(R_m-1)N(t)\right)}{A(t)} w_{F_R} k\left[mH(t)\left(p_{i-1,j,k}(t) - p_{i,j,k}(t)\right) + \frac{M(t)}{2}\left(p_{i,j-1,k}(t) - p_{i,j,k}(t)\right)\right]$$

$$+ \frac{\left(R_m-(R_m-1)N(t)\right)}{A(t)}\left[F(t)\left(w_{H_R}i + w_{M_R}j\right)\left(p_{i,j,k-1}(t) - p_{i,j,k}(t)\right) + w_{F_R}\left((1-m)H(t) + \frac{M(t)}{2}\right)\left((k-1)p_{i,j,k-1}(t) - k\,p_{i,j,k}(t)\right)\right] + \frac{2uN_0(1-m)\left(R_m-(R_m-1)N(t)\right)F(t)H(t)}{A(t)}\left[p_{i,j,k-1}(t) - p_{i,j,k}(t)\right]$$

We now introduce the probability generating function G (see [5]), defined as:

$$G(t,x,y,z) = \sum_{i=0}^{\infty}\sum_{j=0}^{\infty}\sum_{k=0}^{\infty} x^i y^j z^k\, p_{i,j,k}(t)$$

where x, y, z correspond to resistance heterozygotes H_R, M_R, F_R, and the absolute values of x, y, z are ≤ 1. The Kolmogorov equations above, of which there are infinite number, transform into a single first-order PDE in four independent variables for the probability generating function $G(t,x,y,z)$:

$$-G\,\frac{2uN_0(1-m)\left(R_m-(R_m-1)N(t)\right)F(t)H(t)}{A(t)}(z-1) = -\frac{\partial G}{\partial t} + \frac{\partial G}{\partial x}\left[(1-x) + \frac{w_{H_R}\left(R_m-(R_m-1)N(t)\right)F(t)}{A(t)}x(z-1)\right] + \frac{\partial G}{\partial y}\left[(1-y) + \frac{w_{M_R}\left(R_m-(R_m-1)N(t)\right)F(t)}{A(t)}y(z-1)\right] + \frac{\partial G}{\partial z}\left[(1-z) + \frac{w_{F_R}\left(R_m-(R_m-1)N(t)\right)}{A(t)}z\left(mH(t)(x-1) + \frac{M(t)}{2}(y-1) + \left((1-m)H(t) + M(t)/2\right)(z-1)\right)\right] \quad \text{(A2.4)}$$

$G(t=0,x,y,z) = x^i y^j z^k$ corresponds to an initial condition of i, j and k individuals of type H_R, M_R and F_R, respectively. The probability of there being no mutants present at time t is given by $p_{0,0,0}(t) = G(t, x=0, y=0, z=0)$, and to obtain the probabilities of interest, it is this quantity that is required rather than the general solution of (A2.4) for all x, y and z. The inhomogeneous transport PDE (A2.4) for $G(t,0,0,0)$ is therefore solved by following the Method of Characteristics [5, 6]. We introduce the parametric curves $x = x(s)$, $y = y(s)$ and $z = z(s)$ for $s \in [0,t]$, define $q(s) := G(t-s, x(s), y(s), z(s))$, and differentiate $q(s)$ with respect to s:

$$\frac{dq(s)}{ds} = -\frac{\partial G(t-s,x(s),y(s),z(s))}{\partial t} + \frac{\partial G(t-s,x(s),y(s),z(s))}{\partial x}\left[\frac{dx(s)}{ds}\right] + \frac{\partial G(t-s,x(s),y(s),z(s))}{\partial y}\left[\frac{dy(s)}{ds}\right] + \frac{\partial G(t-s,x(s),y(s),z(s)}{\partial z}\left[\frac{dz(s)}{ds}\right] \quad \text{(A2.5)}$$

Upon setting $G \rightarrow G(t-s, x(s), y(s), z(s))$, $x \rightarrow x(s)$, $y \rightarrow y(s)$ and $z \rightarrow z(s)$ in (A2.4), the resulting right-hand side of (A2.4) and that of (A2.5) become the same if $x(s), y(s)$ and $z(s)$ satisfy a family of non-linear ODEs, for a given time t (with the driving Y released at $t = 0$):

$$\frac{dx(s)}{ds} = 1 - x(s) + \frac{w_{H_R}\left(R_m-(R_m-1)N(t-s)\right)F(t-s)}{A(t-s)}x(s)(z(s)-1)$$

$$\frac{dy(s)}{ds} = 1 - y(s) + \frac{w_{M_R}\left(R_m-(R_m-1)N(t-s)\right)F(t-s)}{A(t-s)}y(s)(z(s)-1)$$

$$\frac{dz(s)}{ds} = 1 - z(s) + \frac{w_{F_R}\left(R_m-(R_m-1)N(t-s)\right)}{A(t-s)}z(s)\left[mH(t-s)(x(s)-1) + \frac{M(t-s)}{2}(y(s)-1) + \frac{2(1-m)H(t-s)+M(t-s)}{2}(z(s)-1)\right] \quad \text{(A2.6)}$$

These key equations are solved numerically using Wolfram Mathematica NDSolve [3]. An initial condition $x(s=0) = y(s=0) = z(s=0) = 0$ is specified which corresponds to $G(t, x=0, y=0, z=0) = p_{0,0,0}(t)$, i.e. the probability that no mutants are present at time t. Note that if male mutant fitnesses are equal, $w_{M_R} = w_{H_R} = w_{A_R}$, with the corresponding branching equations given by Eq. (A2.3), one obtains only two equations to be solved simultaneously, with $y(s)$ corresponding to the total male mutants, $A_R = H_R + M_R$, and $z(s)$ to female mutants F_R:

$$\frac{dy(s)}{ds} = 1 - y(s) + \frac{w_{A_R}\left(R_m-(R_m-1)N(t-s)\right)F(t-s)}{A(t-s)}y(s)(z(s)-1)$$

$$\frac{dz(s)}{ds} = 1 - z(s) + \frac{w_{F_R}\left(R_m-(R_m-1)N(t-s)\right)}{A(t-s)}z(s)\left[\left(\frac{2mH(t-s)+M(t-s)}{2}\right)(y(s)-1) + \frac{2(1-m)H(t-s)+M(t-s)}{2}(z(s)-1)\right]$$

Returning to the general case, with $x(s), y(s)$ and $z(s)$ specified by (A2.6), we set $G \rightarrow G(t-s, x(s), y(s), z(s))$, $x \rightarrow x(s)$, $y \rightarrow y(s)$ and $z \rightarrow z(s)$ in (A2.4) and equate the left-hand-sides of (A2.4) and (A2.5) to yield:

$$\frac{dq(s)}{ds} = \frac{2uN_0(1-m)\left(R_m-(R_m-1)N(t-s)\right)F(t-s)H(t-s)}{A(t-s)}\left(1-z(s)\right)G(t-s,x(s),y(s),z(s))$$

$$= \frac{2uN_0(1-m)\left(R_m-(R_m-1)N(t-s)\right)F(t-s)H(t-s)}{A(t-s)}\left(1-z(s)\right)q(s) \quad \text{(A2.7)}$$

This equation is integrated from $s = 0$ to $s = t - t_a$. Since s is effectively reverse time from the current time t, these integration limits correspond respectively to time t and to an initial time that is specified as t_a for generality (i.e., $t_a = 0$ for starting time corresponding to the time of release of the driving Y male, and a starting time of $t_a \geq 0$ corresponding to a number of mutant(s) introduced at time t_a in the absence of mutation). Integration of (A2.7) yields:

$$q(s=0) = q(s=t-t_a)\exp\left[-2uN_0(1-m)\int_0^{t-t_a}\frac{\left(R_m-(R_m-1)N(t-s)\right)F(t-s)H(t-s)}{A(t-s)}\left(1-z(s)\right)ds\right]$$

The definition $q(s) := G(t-s, x(s), y(s), z(s))$ thus yields:

$$G(t,0,0,0) = G\left(t_a, x(t-t_a), y(t-t_a), z(t-t_a)\right) \times \\ \exp\left[-2uN_0(1-m)\int_0^{t-t_a}\frac{\left(R_m-(R_m-1)N(t-s)\right)F(t-s)H(t-s)}{A(t-s)}\left(1-z(s)\right)ds\right] \quad \text{(A2.8)}$$

This is a general solution for the probability of no mutants present at time t with driving Y release at $t=0$. The first factor represents the initial condition of an arbitrary number i, j, k (could be zero) of pre-existing mutants of type H_R, M_R or F_R present at $t=t_a$, given by $G\left(t_a, x(t-t_a), y(t-t_a), z(t-t_a)\right) = x^i y^j z^k$; the second (exponential) factor represents the contribution of cleavage-induced mutations ($u \neq 0$) from time t_a to t.

Introduction of single mutation, $p_{est}(t_a)$. The focus is first on the case of an individual resistant mutant of type H_R, M_R or F_R, that is "introduced" at time t_a after the release of the driving Y at $t=0$, in the absence of cleavage-induced mutation ($u=0$). For $u=0$ (i.e., $r_{Mut}=0$), the exponential expression in (A2.8) becomes one, leaving the initial condition of i, j, k of mutants of each type H_R, M_R, F_R present at time t_a:

$$G(t,0,0,0) = G(t_a, x(t-t_a), y(t-t_a), z(t-t_a)) = x(t-t_a)^i y(t-t_a)^j z(t-t_a)^k$$

So, the probability of at least one mutant of any type being present at time is:

$$1-p_{0,0,0}(t) = 1-G(t,0,0,0) = 1-x(t-t_a)^i y(t-t_a)^j z(t-t_a)^k$$

To obtain the probability that a single mutant introduced at time t_a survives stochastic loss and one or more mutants (of any type) are present in the population at time t, we set $i, j, k = 1,0,0$ for an introduction of one H_R individual, $i, j, k = 0,1,0$ for an introduction of one M_R individual, and $i, j, k = 0,1,1$ for an introduction of one F_R individual. The ultimate probability of establishment ($t \to \infty$) of a mutant introduced as a single individual of type H_R, M_R or F_R at time t_a is thus denoted by $p_{est,H_R}(t_a), p_{est,M_R}(t_a)$ or $p_{est,F_R}(t_a)$, and given by:

$$p_{est,H_R}(t_a) = \lim_{t\to\infty}[1-x(t-t_a)]$$

$$p_{est,M_R}(t_a) = \lim_{t\to\infty}[1-y(t-t_a)]$$

$$p_{est,F_R}(t_a) = \lim_{t\to\infty}[1-z(t-t_a)] \quad \text{(A2.9)}$$

These equations for multiple mutant types, with x, y, z given by numerical solution of (A2.6), are the analogous expressions to Uecker and Hermisson's Eq. (16) [4] for $p_{est}(t_a)$ for a single mutant type; a closed-form integral solution is obtainable for the latter simpler case [4, 5].

Probability of mutation arising and surviving, P_1. The focus is now on recovery of the population due to establishment of mutants created by cleavage-induced mutation with nonzero mutation parameter u, and no pre-existing mutation. The probability that least one mutant is present at time t is $p_1(t)$, and

$P_1 = p_1(t \to \infty)$ is the probability that at least one mutation has arisen and has survived stochastic loss and the population therefore persists indefinitely. Since it is specified that there are no resistant mutants introduced at any time t_a, the initial condition (i.e. the first factor in (A2.8)) is simply (for any time t_a): $G\big(t_a, x(t-t_a), y(t-t_a), z(t-t_a)\big) = x(t-t_a)^0 y(t-t_a)^0 z(t-t_a)^0 = 1$, leaving only the exponential factor in (A2.8) for cleavage-induced mutations that can arise at any time after introduction of the driving Y ($t = 0$), hence the integration limits from 0 to t:

$$G(t,0,0,0) = \exp\left[-2uN_0(1-m)\int_0^t \frac{\big(R_m-(R_m-1)N(t-s)\big)F(t-s)H(t-s)}{A(t-s)}\big(1-z(s)\big)\,ds\right] \qquad \text{(A2.10)}$$

The probability $p_1(t)$ that at least one resistant allele is present at time t after introduction of the driving Y is given by: $p_1(t) = 1 - p_{0,0,0}(t) = 1 - G(t,0,0,0)$. From (A2.10), and transforming the integration variable as $s \to t - \tau$, we obtain:

$$p_1(t) = 1 - \exp\left[-2uN_0(1-m)\int_0^t \frac{\big(R_m-(R_m-1)N(\tau)\big)F(\tau)H(\tau)}{A(\tau)}\big(1-z(t-\tau)\big)\,d\tau\right]$$

The probability P_1 that at least one mutation will arise and establish over all time is $p_1(t \to \infty)$, and substituting $\lim_{t\to\infty}\big(1-z(t-\tau)\big) = p_{est,F_R}(t_a)$ from (A2.9) above yields:

$$P_1 = 1 - \exp\left[-2uN_0(1-m)\int_0^\infty \frac{\big(R_m-(R_m-1)N(\tau)\big)F(\tau)H(\tau)}{A(\tau)}\,p_{est,F_R}(\tau)\,d\tau\right]$$

This is Eq. (3) in the main text, with $p_{est,F_R}(\tau)$ calculated from (A2.9) and following the solution of (A2.6). Note that only $p_{est,F_R}(\tau)$ (derived from the solution for $z(s)$ corresponding to F_R mutants) appears in the integral, because X chromosome resistant mutations can only spontaneously arise in daughters of driving-Y males; however, it is still necessary to solve the coupled equations (A2.6) for all quantities $x(s), y(s)$ and $z(s)$. The probability that at least one successful mutation will arise is the rate of creation of the mutant $r_{Mut}(\tau)$ over all times, weighted by the time-varying probability of establishment of a mutant F_R arising at time τ, $P_{est,F_R}(\tau)$ (see Eq. (A7) in [4]).

Probability of mutation P_{Mut}. It is assumed that no mutations are present before the driving Y is introduced, but subsequently cleavage-induced mutations can first occur in female progeny from driving Y males at a rate $r_{Mut}(t)$, $(u \neq 0)$. The probability that at least one resistant X has been created naturally (for $u > 0$) by time t is denoted as $p_{Mut}(t)$, and for a nonhomogeneous Poisson process it is given by:

$$p_{Mut}(t) = 1 - \exp\left[-\int_0^t r_{Mut}(\tau)\,d\tau\right] \qquad \text{(A2.11)}$$

where $r_{Mut}(t)$ is the time-dependent population-wide rate at which F_R individuals arise by mutation (A2.2). Cleavage-induced mutations can arise at any time between introduction of the driving Y and total population extinction. $P_{Mut} = p_{Mut}(t \to \infty)$ is defined as the probability that at least one mutation occurs before the population is extinguished (the mutation may subsequently have gone extinct, or survived and fixed). Eq. (A2.11) then becomes Eq. (5) in the main paper:

$$P_{Mut} = 1 - \exp\left[-2(1-m)(uN_0)\int_0^\infty \frac{\big(R_m-(R_m-1)N(\tau)\big)F(\tau)H(\tau)}{A(\tau)}\,d\tau\right]$$

where two times the integral above,

$$A = 2\int_0^\infty \frac{(R_m - (R_m - 1)N(\tau))F(\tau)H(\tau)}{A(\tau)}\,d\tau \tag{A2.12}$$

represents the total number of progeny from driving Y fathers after release and before elimination in the absence of resistant mutations, of which $(1-m)A$ are females and mA are driving Y males, normalized by N_0.

Exemplar results for these time-dependent probabilities for baseline parameters are presented in Fig. A2.1. Fig. A2.2 shows the variation of $p_{est,F_R}(t_a)$ with the intrinsic growth R_m and with m (strength of Y drive).

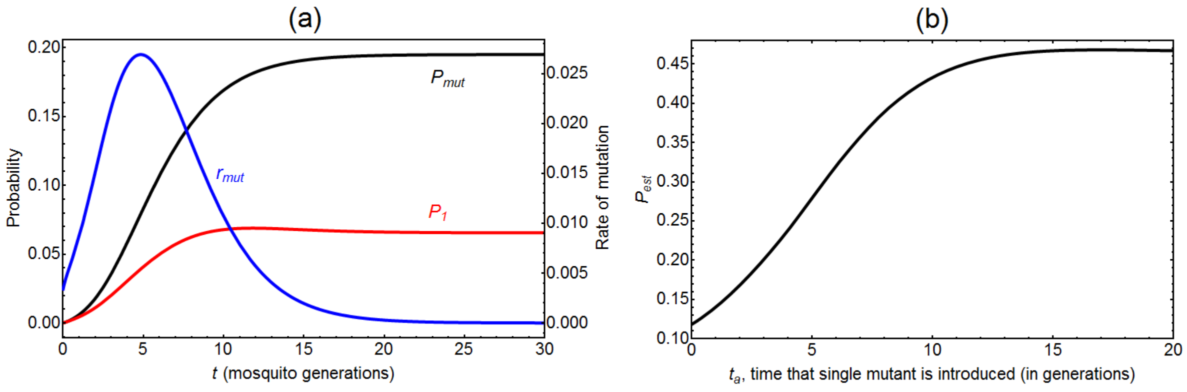

Figure A2.1. (a) Time evolution of $r_{Mut}(t)$, $p_{Mut}(t)$, and $p_1(t)$ for a resistant X chromosome. The probability $p_1(t)$, that at least one mutant has arisen and is present at time t (calculated using the branching process), is lower than $p_{Mut}(t)$, because a proportion of mutants that arise will not survive due to stochastic loss. The long-term limit of $p_1(t)$ is P_1, the probability that at least one successful mutation occurs, spreads, and the total population recovers. (b) Probability of establishment $p_{est,F_R}(t_a)$ of a single new mutation in a female, F_R, introduced at time t_a after driving Y release at $t = 0$, which is higher if it arises at later times t_a after driving Y introduction (up to a plateau for baseline parameters). At later times, when the population is declining and driving Y frequency is high, the recruitment rate is higher due to less density-dependent competition, and consequently a newly-arisen female mutant has a higher probability of passing on the mutation (and passing it to a Y drive male offspring) before dying, and thus the resistant allele has a greater probability of establishing, although less than 50%. For $R_m = 6, m = 0.95, uN_0 = 1, h_0 = 0.05, w = 1$.

The probability of establishment of a single copy of the mutation, $p_{est,F_R}(t_a)$, is affected by the intrinsic rate of increase of the population, R_m, and the transmission rate of the driving Y, m (Fig. A2.2). Overall, $p_{est,F_R}(t_a)$ increases with increasing R_m, and decreases as m increases for the values investigated, most markedly for mutations introduced at later times t_a.

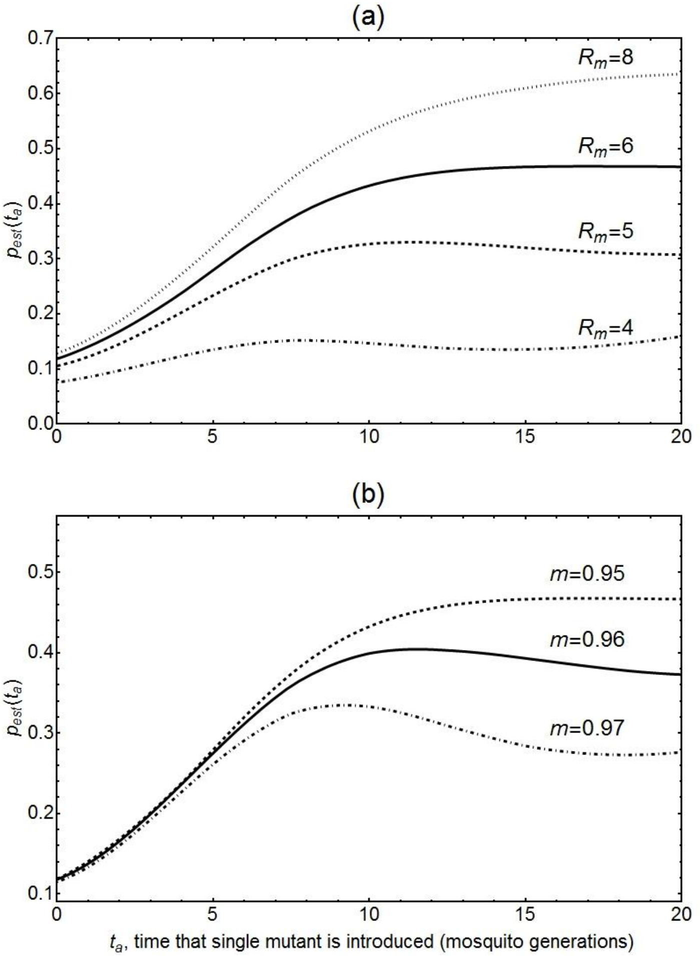

Figure A2.2. Probability of establishment, $p_{est,F_R}(t_a)$, of a single cost-free resistant X-chromosome mutation (Model I) in a female mutant introduced at time t_a (after driving Y release at $t = 0$). (a) For different values of the intrinsic growth R_m and $m = 0.95$. The probability is low initially and increases for mutations that arise at later times (in the absence of any other mutation). The increase in $p_{est,F_R}(t_a)$ with R_m is greater at later times of arising t_a, because the recruitment rate $R_m - (R_m - 1)N(t)$ varies with time after driving Y introduction from about 1 to R_m. (b) For different values of m (strength of Y drive) and $R_m = 6$. Interestingly, the stronger the drive, the lower the probability at later times. This is because the stronger drive produces a more extreme male bias, which in turn means the expected reproductive success of an individual resistant male will be lower (due to the greater relative scarcity of females to mate with), and therefore the overall probability of mutant establishment will be lower. For both plots, $h_0 = 0.05, uN_0 = 1, w = 1$.

A2.2 Model II

The same linearization method as for the resistant model is followed to construct a branching process for the resistant types based on the birth and death events from (A1.8):

$$H_S \to H_S + 1 \qquad \big(R_m - (R_m - 1)N(t)\big)\left[\frac{mH(t)}{A(t)} w_{F_S} F_S + \frac{F(t)}{2A(t)} w_{H_S} H_S\right] + \frac{4vN_0\big(R_m-(R_m-1)N(t)\big)F(t)mH(t)}{A(t)}$$

$$M_S \to M_S + 1 \qquad \big(R_m - (R_m - 1)N(t)\big)\left[\frac{M(t)}{2A(t)} w_{F_S} F_S + \frac{F(t)}{2A(t)} w_{M_S} M_S\right] + \frac{2vN_0\big(R_m-(R_m-1)N(t)\big)F(t)M(t)}{A(t)}$$

$F_S \to F_S + 1$ $\quad$ $\left(R_m - (R_m - 1)N(t)\right)\left[\left(\frac{M(t)}{2A(t)} + \frac{(1-m)H(t)}{A(t)}\right) w_{F_S} F_S + \frac{F(t)}{2A(t)} w_{M_S} M_S + \frac{F(t)}{2A(t)} w_{H_S} H_S\right] + 2vN_0\left(R_m - (R_m - 1)N(t)\right)F(t)\left(\frac{M(t)}{A(t)} + \frac{2(1-m)H(t)}{A(t)}\right)$

$H_S \to H_S - 1$ $\quad$ H_S

$M_S \to M_S - 1$ $\quad$ M_S

$F_S \to F_S - 1$ $\quad$ F_S

Note that if it is assumed that $w_{M_S} = w_{H_S} = w_{A_S}$, the expressions above simplify to:

$A_S \to A_S + 1$ $\quad$ $\left(R_m - (R_m - 1)N(t)\right)\left[\frac{(2mH(t)+M(t))}{A(t)} w_{F_S} F_S + \frac{F(t)}{2A(t)} w_{A_S} A_S\right] + \frac{2vN_0\left(R_m-(R_m-1)N(t)\right)F(t)(2mH(t)+M(t))}{A(t)}$

$F_S \to F_S + 1$ $\quad$ $\left(R_m - (R_m - 1)N(t)\right)\left[\left(\frac{M(t)}{2A(t)} + \frac{(1-m)H(t)}{A(t)}\right) w_{F_S} F_S + \frac{F(t)}{2A(t)} w_{A_S} A_S\right] + 2vN_0\left(R_m - (R_m - 1)N(t)\right)F(t)\left(\frac{M(t)}{A(t)} + \frac{2(1-m)H(t)}{A(t)}\right)$

$A_S \to A_S - 1$ $\quad$ A_S

$F_S \to F_S - 1$ $\quad$ F_S

Returning to the general case, we follow a similar procedure as above and introduce the probability generating function $G(t, x, y, z)$, where x, y, z correspond to H_S, M_S, F_S heterozygotes:

$$\frac{\partial G}{\partial t} = G\frac{2vN_0\left(R_m-(R_m-1)N(t)\right)F(t)}{A(t)}\left[2mH(t)(x-1) + M(t)(y-1) + \left(M(t) + 2(1-m)H(t)\right)(z-1)\right] + \frac{\partial G}{\partial x}\left[(1-x) + \frac{w_{H_S}\left(R_m-(R_m-1)N(t)\right)F(t)}{A(t)}x(x+z-2)\right] + \frac{\partial G}{\partial y}\left[(1-y) + \frac{w_{M_S}\left(R_m-(R_m-1)N(t)\right)F(t)}{A(t)}y(y+z-2)\right] + \frac{\partial G}{\partial z}\left[(1-z) + \frac{w_{F_S}\left(R_m-(R_m-1)N(t)\right)}{A(t)}z\left(mH(t)(x-1) + \frac{M(t)}{2}(y-1) + \left((1-m)H(t) + \frac{M(t)}{2}\right)(z-1)\right)\right] \quad \text{(A2.13)}$$

Introduction of single mutation, $p_{est}(t_a)$. For the probabilities of establishment $p_{est,H_S}(t_a)$, $p_{est,M_S}(t_a)$, $p_{est,F_S}(t_a)$ of mutant H_S, M_S, F_S, we proceed as described above for Model I, using the Method of Characteristics to yield equations for $x(s), y(s), z(s)$:

$$\frac{dx(s)}{ds} = 1 - x(s) + \frac{w_{H_S}\left(R_m-(R_m-1)N(t-s)\right)F(t-s)}{A(t-s)}x(s)\left(x(s) + z(s) - 2\right)$$

$$\frac{dy(s)}{ds} = 1 - y(s) + \frac{w_{M_S}\left(R_m-(R_m-1)N(t-s)\right)F(t-s)}{A(t-s)}y(s)\left(y(s) + z(s) - 2\right)$$

$$\frac{dz(s)}{ds} = 1 - z(s) + \frac{w_{F_S}\left(R_m - (R_m - 1)N(t-s)\right)}{A(t-s)} z(s)\left(mH(t-s)(x(s)-1) + \frac{M(t-s)}{2}(y(s)-1) + \left((1-m)H(t-s) + \frac{M(t-s)}{2}\right)(z(s)-1)\right) \quad \text{(A2.14)}$$

Note that if it is assumed that $w_{M_S} = w_{H_S} = w_{A_S}$, we obtain:

$$\frac{dy(s)}{ds} = 1 - y(s) + \frac{w_{A_S}\left(R_m - (R_m - 1)N(t-s)\right) F(t-s)}{A(t-s)} y(s)(y(s) + z(s) - 2)$$

$$\frac{dz(s)}{ds} = 1 - z(s) + \frac{w_{F_S}\left(R_m - (R_m - 1)N(t-s)\right)}{A(t-s)} z(s)\left(\frac{2mH(t-s) + M(t-s)}{2}(y(s)-1) + \left((1-m)H(t-s) + \frac{M(t-s)}{2}\right)(z(s)-1)\right)$$

These equations are solved numerically using Wolfram Mathematica NDSolve, with initial conditions $x(s=0) = y(s=0) = z(s=0) = 0$. Following the same method as above, the probability of establishment ($t \to \infty$) of a mutant present as a single individual of type $H_S(i,j,k = 1,0,0)$, $M_S(i,j,k = 0,1,0)$ or $F_S(i,j,k = 0,0,1)$ at $t = t_a$ is then given by:

$$p_{est,H_S}(t_a) = \lim_{t\to\infty}[1 - x(t - t_a)]$$

$$p_{est,M_S}(t_a) = \lim_{t\to\infty}[1 - y(t - t_a)]$$

$$p_{est,F_S}(t_a) = \lim_{t\to\infty}[1 - z(t - t_a)] \quad \text{(A2.15)}$$

Probability of mutation arising and surviving, P_1. Again using the Method of Characteristics, (A2.13) is solved for $G(t,0,0,0)$ with $v \neq 0$ and initial condition $G(t=0,x,y,z) = 1$. P_1 is the probability that at least one female or male heterozygote will arise, establish, and lead to population recovery:

$$P_1 = 1 - \exp\left[-2(vN_0)\int_0^\infty \frac{\left(R_m - (R_m-1)N(\tau)\right)F(\tau)}{A(\tau)}\left(2m\,H(\tau)\,[\lim_{t\to\infty}\left(1 - x(t-\tau)\right)] + M(\tau)\,[\lim_{t\to\infty}\left(1 - y(t-\tau)\right)] + \left(M(\tau) + 2(1-m)H(\tau)\right)[\lim_{t\to\infty}\left(1 - z(t-\tau)\right)]\right) d\tau\right]$$

From (A2.15) above we substitute to obtain Eq. (4) in the main paper:

$$P_1 = 1 - \exp\left[-2(vN_0)\int_0^\infty \frac{\left(R_m - (R_m-1)N(\tau)\right)F(\tau)}{A(\tau)}\left(2mH(\tau)\,p_{est,H_S}(\tau) + M(\tau)p_{est,M_S}(\tau) + \left(M(\tau) + 2(1-m)H(\tau)\right)p_{est,F_S}(\tau)\right) d\tau\right]$$

Note that a trans-acting suppressor mutation can arise naturally in any mutant type, therefore the probabilities of establishment of all three types are included in the expression above, while for the X chromosome mutation, only $p_{est,F_S}(\tau)$ appears.

Probability of mutation P_{Mut}. Unlike new target-resistant X-chromosome mutations which appear only in female progeny of driving Y males, for the suppressor mutation, every birth from non-mutants has the potential for a new suppressor mutation to arise on an autosome. The total combined rate at which H_S, M_S and F_S individuals first arise is given by:

$$r_{Mut}(t) = r_{Mut,H_S}(t) + r_{Mut,M_S}(t) + r_{Mut,F_S}(t)$$

$$= \frac{4vN_0\left(R_m-(R_m-1)N(t)\right)F(t)mH(t)}{A(t)} + \frac{2vN_0\left(R_m-(R_m-1)N(t)\right)F(t)M(t)}{A(t)} + \frac{2vN_0\left(R_m-(R_m-1)N(t)\right)F(t)[M(t)+2(1-m)H(t)]}{A(t)}$$

$$= 4vN_0\left(R_m-(R_m-1)N(t)\right)F(t) \qquad \text{(A2.16)}$$

The time evolution of the rates at which H_S, M_S and F_S individuals first arise, and their total, is shown in Fig. A2.3 for baseline parameters, along with the mutation rate for the resistant X-chromosome mutation (with v and u chosen to give the same total probability P_{Mut} that a mutation occurs before population extinction, to facilitate comparison).

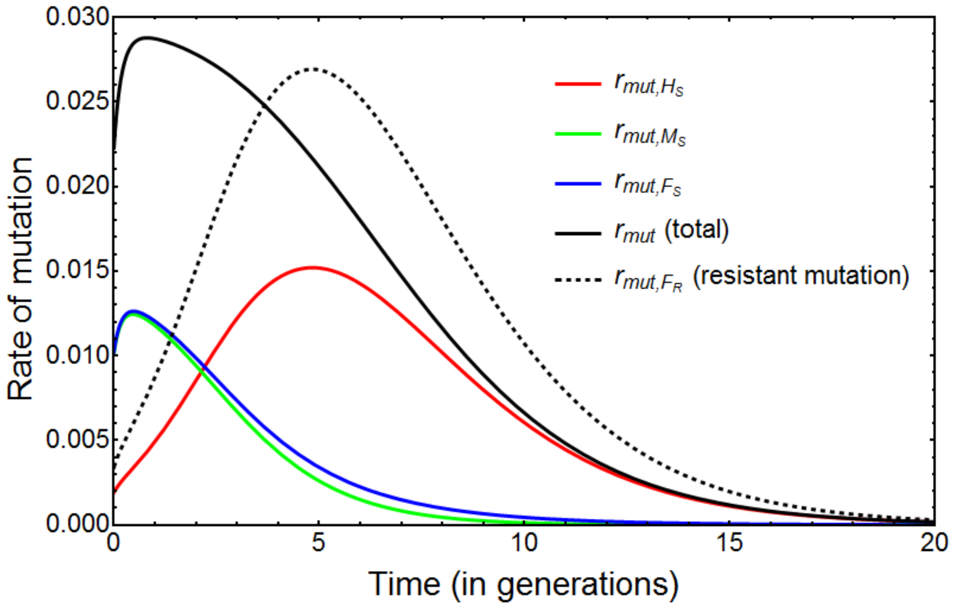

Figure A2.3. Time evolution of the population-wide rates $r_{Mut}(t)$ that new suppressor mutations arise in driving Y males H_S (red line) and wild-type males M_S (green line), females F_S (blue line), and in total (black line), for $R_m = 6, m = 0.95, h_0 = 0.05, w = 1$, and $vN_0 = 0.0149$ chosen for comparison, such that overall probability that a mutation will arise before extinction is the same for both, $P_{Mut} = 0.195$. Initially, mutations mainly occur in wild-type females and males; later in driving Y males. Also shown (dotted line) is $r_{Mut}(t)$ for the resistant mutation (arising in females only, F_R), for $uN_0 = 1$.

The total probability that at least one mutation (in a female, male or driving Y male) will have arisen by time t is given by substituting the rate $r_{Mut}(t)$ above into (A2.11):

$$p_{Mut}(t) = 1 - \exp\left[-4(vN_0)\int_0^t (R_m - (R_m-1)N(\tau))F(\tau)\,d\tau\right]$$

We then arrive at (6) in the main paper, which is the probability of at least one mutation having arisen before the population is eliminated:

$$P_{Mut} = 1 - \exp\left[-4(vN_0)\int_0^\infty (R_m - (R_m - 1)N(\tau))F(\tau)\,d\tau\right] \quad \text{(A2.17)}$$

Above, the integral, times a factor of two, represents the total number of progeny of all types after release and before elimination if no resistance evolves, normalized by N_0.

Exemplar plots of the time-evolution of $r_{Mut}(t)$, $p_{Mut}(t), p_1(t)$, and $p_{est}(t_a)$ for baseline parameters are shown in Fig. A2.4.

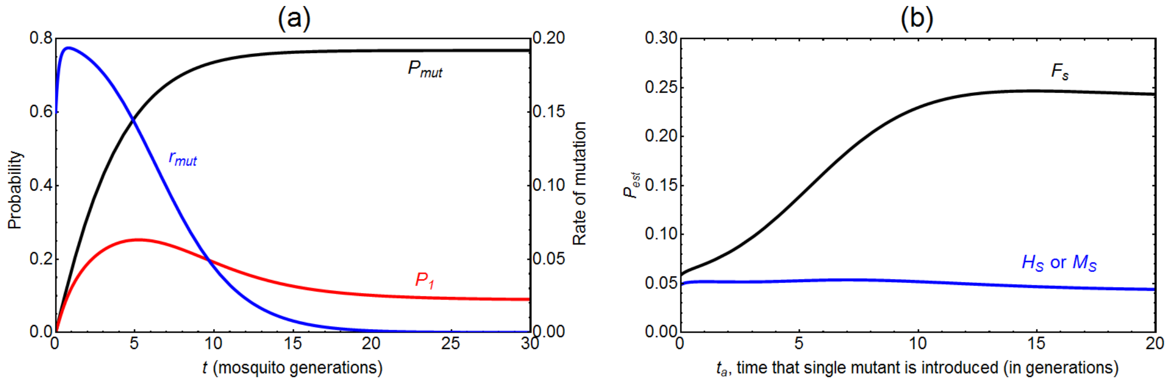

Figure A2.4. (a) Time evolution of $r_{Mut}(t)$, $p_{Mut}(t)$, and $p_1(t)$ for the suppressor mutation, calculated via the branching process method. The long-term limit P_1 is the probability that at least one successful mutation occurs, spreads, and the total population recovers. (b) Time evolution of probability of establishment $p_{est}(t_a)$ for a single new suppressor mutation at time t_a, in a heterozygote female (black line) and in a heterozygote male, driving Y or wild-type (blue line), assuming that wild-type and driving Y male fitnesses are the same. For these parameters, the probability of survival for new mutations arising in a female at time t_a rises and plateaus, but for those arising in a male it does not change significantly. Similarly to the resistant mutation model, declining population at later times means less density-dependent competition and thus higher chance of a female mutant establishing, while the higher male sex bias at later times results in male mutants making a decreased per-capita contribution to passing on the mutation, and thus males have a lower probability of establishment than female mutants. For $R_M = 6, m = 0.95, h_0 = 0.05, vN_0 = 0.1, w = 1$.

A2.3 Applicability of branching process method

Second-order terms and homozygote populations are ignored in the linearization of the equations that leads to the branching process formulation, but since early stochastic loss generally happens when mutant populations are small, this approximation usually holds for calculating the probability that a mutation establishes. However, for our equations with time-varying rates, for certain parameters, the solution of the linearized equations is zero at long times after driving Y release, and thus growing mutant populations will eventually fall back to zero before the probability of stochastic loss has converged. Here, the branching process cannot be used, and full simulations are used to calculate the probabilities of the mutation surviving stochastic loss (see Section A3 below). In all other regions, the computationally faster and more convenient branching process equations are used and values are confirmed with simulations as needed.

Model I. To find the parameter space where linearization for the resistant X mutation cannot be used, we consider equations (A1.2), linearized and in the long-time limit, where it can be assumed that $R_m \gg \gamma N(t)$, $M, F, H, M_R \cong 0$ and $F(t \to \infty)/H(t \to \infty) \cong (1-m)/m$. While the linearization approach implicitly assumes that once a mutant population is established, it will continue growing, there exists a range of parameters for which the solutions of these equations eventually decay with time such that $F_R(t \to \infty) = H_R(t \to \infty) = 0$ (for supercritical $m \geq m_{crit} = 1 - \frac{1}{2 R_m}$). Such non-monotonic solutions occur if the following condition (for $w_{F_R} = w$ and $w_{H_R} = w^2$) is satisfied:

$$(1-m)\, R_m\, w\, (1 + R_m w^2) < 1$$

This condition can be also written either in terms of m or R_m:

$$-\frac{1}{2w^2} + \frac{1}{2w^2}\sqrt{\frac{1-m+4w}{1-m}} > R_m \quad \text{or} \quad 1 - \frac{1}{w^3 {R_m}^2 + w R_m} < m$$

The equations for the branching process are thus less likely to hold at conditions of low R_m, high m, and/or high cost to the mutation.

Model II. As for Model I above, we consider the long-time behaviour of the linearized equations for the heterozygotes for the trans-acting suppressor mutation, derived from the nonlinear equations (A1.8). The conditions for which the branching process model cannot be used for the suppressor mutation (for $w_{H_S} = w_{F_S} = w$) are:

(i) for $> \frac{-2+3R_m+\sqrt{(4-8R_m+5{R_m}^2+{R_m}^3)/(1+R_m)}}{4R_m}$, it does not hold for any w;

(ii) for $\leq \frac{-2+3R_m+\sqrt{(4-8R_m+5{R_m}^2+{R_m}^3)/(1+R_m)}}{4R_m}$, it does not hold for

$$0 < w \leq \frac{1+2m+\sqrt{[1-m(5-4(4-m)m)]/(1-m)}}{2(1-2m)R_m}.$$

Again, for conditions of low R_m, high m, and/or high cost to the mutation, the branching process method cannot be used (full simulations are carried out instead).

A3. Simulation method

Stochastic simulations are carried out using the direct Gillespie algorithm. In this approach, $\boldsymbol{Y}(t)$ represent integer numbers of individuals (Model I is considered first, with $\boldsymbol{Y} = \{H, M, F, H_R, M_R, F_R, F_{RR}\}$ and populations of all types vary stochastically. $Y_i(t)$ is the number of individuals of type i ($i = 1{,}7$) at time t and the total number of individuals is $N(t) = \sum_{i=1}^{7} Y_i(t)$. The transition rates are designated according to the basic model (A1.2) for the birth and death of each individual:

$Y_i \to Y_i + 1 \qquad b_i(\boldsymbol{Y})$

$Y_i \rightarrow Y_i - 1$ Y_i

We define propensity functions $\alpha_i(t) = b_i(\boldsymbol{Y}(t))$ and $\alpha_{i+7}(t) = Y_i(t)$ for $i = 1,7$. The waiting time for the next transition, i.e. the next birth or death event of a single individual of any type, is $\tau = -\frac{\log(r_1)}{\alpha_T(t)}$, where $\alpha_T(t) = \sum_{i=1}^{14} \alpha_i(t)$ is the total propensity function and r_1 is a random number uniformly distributed in [0,1]. To decide which event (indexed by $j = 1, 14$) takes place at time $t + \tau$, a second random number r_2 is generated and the index j that satisfies $\sum_{i=0}^{j-1} \frac{\alpha_i(t)}{\alpha_T(t)} < r_2 \leq \sum_{i=1}^{j} \frac{\alpha_i(t)}{\alpha_T(t)}$ (with $\alpha_0(t) = 0$) is chosen. If $1 \leq j \leq 7$, a birth for Y_J has occurred and $Y_J(t + \tau) = Y_J(t) + 1$; if $8 \leq j \leq 14$, a death for Y_{J-7} has occurred and $Y_{J-7}(t + \tau) = Y_{J-7}(t) - 1$. The same steps are repeated until t reaches an upper limit or the populations have reached desired limits (see below). For the suppressor mutation, the same approach is used for the nine different types $F, F_S, F_{SS}, H, H_S, H_{SS}, M, M_S, M_{SS}$ with transition rates based on Eq. (A1.8).

A carrying capacity of equal numbers of wild-type males and females ($M = F = N_0/2$) is specified, typically $N_0 = 10^6$, and driving Y introduction $h_0 = 0.05N_0$ at time zero (a quantity high enough to ensure no early stochastic loss of the driving Y). For calculation of P_1 and P_{Mut}, we specify non-zero u, v, and for calculation of $p_{est}(t_a)$ we set $u, v = 0$ and introduce a single mutation of specified type (female, male or wild-type male, as appropriate) at a given time t_a after driving Y introduction. To find the percentage of runs (total runs $= 10^6$) for which no individuals are present after long times, simulations are run until either (1) the total population is extinct (either no males or no females) or (2) the mutant population reaches the equilibrium level that is predicted by deterministic equations; in practice for considerations of speed, calculations are stopped at a percentage (between 50-80%, but no less than 50,000 total population size) of that level sufficiently high that the mutation can be considered established.

A large number of individuals ($N_0 = 10^6$) is used in simulations in order to confirm the results of the branching process method. The latter assumes a very large baseline population size; the close agreement between the two calculation methods suggests that 10^6 indeed counts as large. For a subset of parameter values, simulations with $N_0 = 10^4$ and 10^5 were also run (for equivalent population-wide mutation rates) and there was little effect on P_1. However, there are still several parameter regions where there are stochastic effects of finite numbers of discrete individuals. Firstly, in a small percentage of runs at very low R_m, all driving Y males are lost around the time when the population is almost eliminated, leaving behind a few wild-type males and females. In some cases, these wild-types re-establish and the wild-type population recovers. Secondly, for conditions of very weak drive (close to m_{crit}), an 'infinite' population will only go to zero in the limit of $t \rightarrow \infty$, but for finite numbers of individuals the population will eventually reach zero (at roughly the time when the normalised population reaches $1/N_0$ in the deterministic models). Thus for a simulation of a finite number of individuals, the probability of a mutation will be expected to be smaller than the limit for an 'infinite' number. As our main interest is in strong drive, this latter stochastic effect does not unduly concern us here, and in any case, it results in a smaller probability of resistance evolving.

For the resistant X-chromosome (Model I) for baseline parameters ($N_0 = 10^6$), exemplar simulation results of randomly selected runs for which the mutation arose, established and the population recovered are shown in Fig. A3.1 along with the solution of the full deterministic equations (A1.2) in the presence of mutation (dashed line). The deterministic equations always predict recovery for these parameters

(cost-free mutation), while the simulations (10^6 runs) and branching process method predict only a 6.5% probability of evolution of resistance and population recovery.

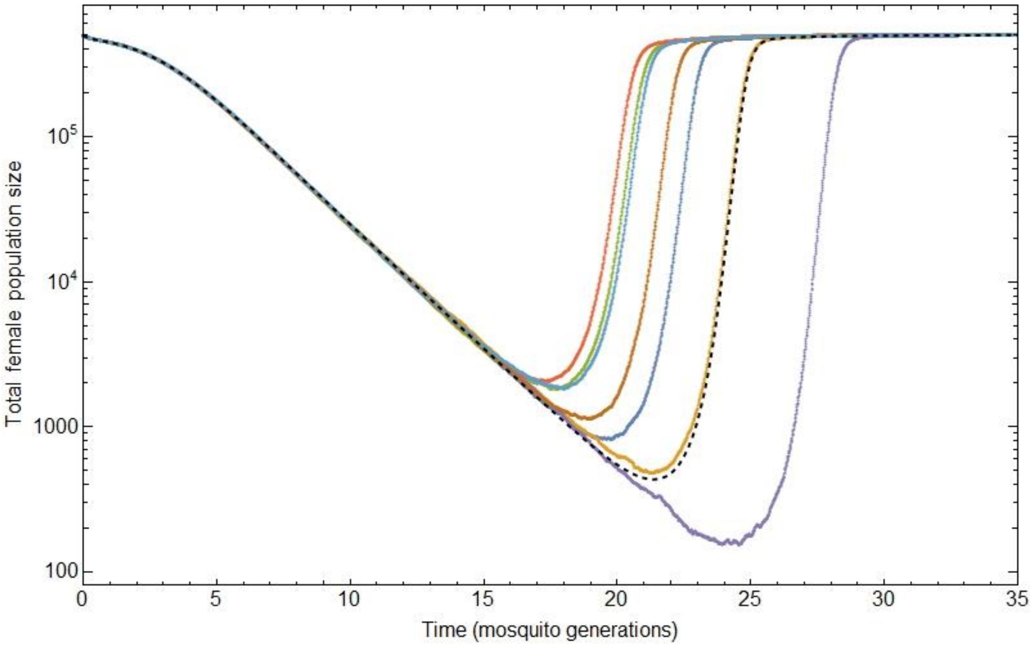

Figure A3.1. Recovery of the total female population in the presence of mutation for Model I for cleavage-induced X chromosome resistance, with driving Y males released at time zero. The coloured data points show randomly selected individual runs from Gillespie simulations for which the population recovered (total 100 runs, of which the 7 shown here had population recovery and the other 93 did not). The dashed line shows the solution of the deterministic equations. For wild-type pre-release population $N_0 = 10^6$, and parameters $R_m = 6, m = 0.95, uN_0 = 1, h_0 = 0.05, w = 1, \gamma = (R_m - 1)/N_0$. For 10^6 runs for these parameters, the simulations give probability of population recovery $P_1 \cong 0.065$, which agrees extremely well with the branching process model ($P_1 \cong 0.065$).

Using the full simulations, the distribution of times that the total female population is suppressed to a given percentage of its pre-driving-Y release value is also investigated, for the resistant X-chromosome (Model I) for baseline parameters (Fig. A3.2). Shown are the distributions of suppression times for two values of m (strength of Y drive): supercritical (population is eliminated in absence of mutation) and subcritical (drive not sufficient to eliminate population).

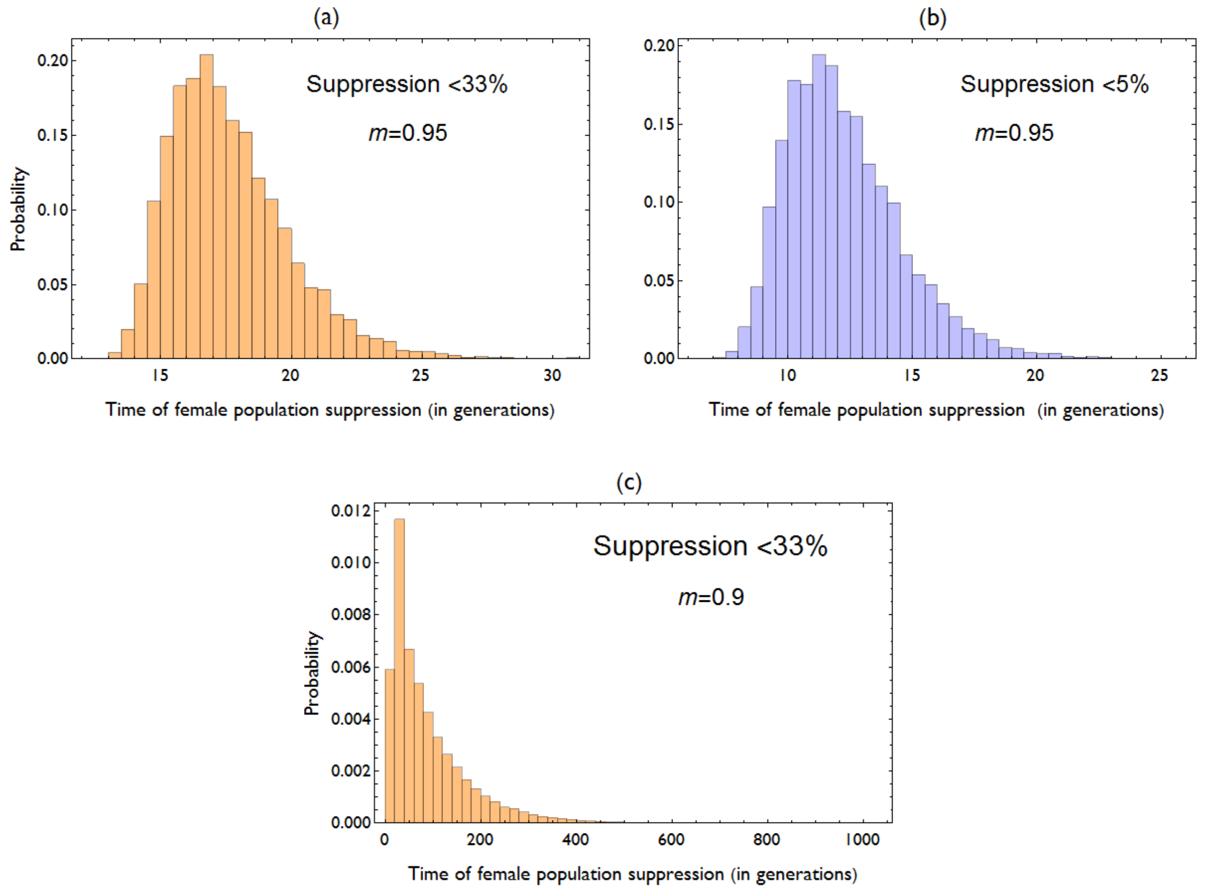

Figure A3.2. Time of suppression of total female population below its pre-driving-Y release value before the resistant X-chromosome mutation (Model 1) rescues the population, calculated from full simulations. Only runs that resulted in population recovery, out of 100,000 total, are shown. (a) Time of suppression < 33%, for $m = 0.95$; mean (median) time is 17.6 (17.3) generations (interquartile range, $16.0 - 18.9$). (b) Time of suppression < 5%, for $m = 0.95$; mean (median) time is 12.3 (11.9) generations (interquartile range, $10.5 - 13.6$). (c) Time of suppression < 33%, for $m = 0.9$; mean (median) time is 90.4 (62.5) generations (interquartile range, $28.2 - 122.0$). Although the probability is too small to be visible on the plot, suppression times of up to 1000 generations occurred. For $R_M = 6, h_0 = 0.05, uN_0 = 1, w = 1$.

References

[1] Beaghton A, Hammond A, Nolan T, Crisanti A, Godfray HCJ, Burt A. Requirements for driving anti-pathogen effector genes into populations of disease vectors by homing. Genetics. 2017;205:1587-96.

[2] Beaghton A, Beaghton PJ, Burt A. Gene drive through a landscape: reaction-diffusion models of population suppression and elimination by a sex ratio distorter. Theor Popul Biol. 2016;108:51–69.

[3] Wolfram Research, Inc., 2015 Mathematica 10.2., Champaign, IL.

[4] Uecker H, Hermisson J. On the fixation process of a beneficial mutation in a variable environment. Genetics 2011;188:915-30.

[5] Allen LJS. An introduction to stochastic processes with applications to biology. 2nd ed. CRC Press; 2010.

[6] Chang CH, Shin J, Junseok KJ. A numerical characteristic method for probability generating functions on stochastic first-order reaction networks. J Math Chem. 2013;51:316–37.
